# Neutron scanning reveals unexpected complexity in the enamel thickness of an herbivorous Jurassic reptile

**DOI:** 10.1098/rsif.2018.0039

**Published:** 2018-06-13

**Authors:** Marc E. H. Jones, Peter W. Lucas, Abigail S. Tucker, Amy P. Watson, Joseph J. W. Sertich, John R. Foster, Ruth Williams, Ulf Garbe, Joseph J. Bevitt, Floriana Salvemini

**Affiliations:** 1 Department of Earth Sciences, The Natural History Museum, London, UK; 2 Department of Genetics and Evolution, School of Biological Sciences, The University of Adelaide, North Terrace, Adelaide, South Australia 5005, Australia; 3 South Australian Museum, North Terrace, Adelaide, South Australia 5001, Australia; 4 Smithsonian Tropical Research Institute, Balboa, Panama; 5 Craniofacial Development and Stem Cell Biology, King's College London, London, UK; 6 Department of Earth Sciences, Denver Museum of Nature and Science, Denver, CO, USA; 7 Museum of Moab, Moab, UT, USA; 8 Department of Adelaide Microscopy, The University of Adelaide, Adelaide, South Australia 5001, Australia; 9 Australian Centre for Neutron Scattering, Australian Nuclear Science and Technology Organisation, Sydney, Australia

**Keywords:** tooth, enamel, dentine, neutron CT, X-ray CT

## Abstract

Eilenodontines are one of the oldest radiation of herbivorous lepidosaurs (snakes, lizards and tuatara) characterized by batteries of wide teeth with thick enamel that bear mammal-like wear facets. Unlike most reptiles, eilenodontines have limited tooth replacement, making dental longevity particularly important to them. We use both X-ray and neutron computed tomography to examine a fossil tooth from the eilenodontine *Eilenodon* (Late Jurassic, USA). Of the two approaches, neutron tomography was more successful and facilitated measurements of enamel thickness and distribution. We find the enamel thickness to be regionally variable, thin near the cusp tip (0.10 mm) but thicker around the base (0.15–0.30 mm) and notably greater than that of other rhynchocephalians such as the extant *Sphenodon* (0.08–0.14 mm). The thick enamel in *Eilenodon* would permit greater loading, extend tooth lifespan and facilitate the establishment of wear facets that have sharp edges for orally processing plant material such as horsetails (*Equisetum*). The shape of the enamel dentine junction indicates that tooth development in *Eilenodon* and *Sphenodon* involved similar folding of the epithelium but different ameloblast activity.

## 1. Introduction

The Rhynchocephalia are today represented by a single living species, the New Zealand tuatara (*Sphenodon punctatus*), but during the Mesozoic they were diverse and widespread [1–6]. In particular, the Eilenodontinae are known from the Mesozoic of South America, North America and Europe [3,7–13]. As the earliest referred members are dated to the Late Triassic [11], eilenodontines potentially represent the oldest radiation of herbivorous lepidosaurs (snakes and lizards + tuatara). They are characterized by deep jaws, broad and closely packed teeth with conspicuous wear facets and unusually thick enamel [7,8]. Their stout teeth possess relatively large bases, apparently suited to withstand high loading and bending forces [1–3]. This dental apparatus was likely used in conjunction with a forward (proal) power stroke to orally process food (chew) prior to swallowing [7,14]. Whereas, in the carnivorous *Sphenodon*, food is cut between longitudinal flanges [14], in eilenodontines the food would likely be cut between the hard enamel edges of opposing wear facets as found in many living mammals [7,15,16]. Because rhynchocephalians have limited or no tooth replacement [10,13,17,18], the resistance of the teeth to fracture and wear is particularly important.

Enamel thickness provides valuable information regarding differences in diet, fracture resistance, developmental history and phylogenetic affinity (e.g. [19–29]). A thicker layer of enamel increases the amount of tooth wear that can be endured and it enables a tooth to apply greater forces to food items before fracture occurs [19,23,29]. Enamel thickness can also indicate the contribution to tooth development made by ameloblast activity rather than folding of the outer enamel epithelium [28,30].

Although enamel thickness has been extensively studied in mammals, particularly primates (e.g. [19,24,27]), quantitative comparisons of enamel thickness among reptiles remain rare [31–34] and are essentially absent for lepidosaurs. The enamel thickness of *Eilenodon* or other eilenodontines has never been specifically measured. In *Sphenodon*, the enamel is considered to be relatively thin and removed fairly rapidly from locations subject to tooth wear (e.g. [14,35]). Estimates of thickness based on mesiodistal sections suggest that it is between 0.07 and 0.13 mm thick [36–38]. Similar sections through the post-hatchling (additional) tooth of a small fossil rhynchocephalian, *Sphenocondor* from the Jurassic of Argentina, suggest an enamel thickness of between 0.03 and 0.04 mm ( [39], fig. 3*d*). In both taxa, the distribution of the enamel broadly resembles that of *Alligator*, with a relatively even distribution that shows some thickening towards the cusp tip [31,32].

Both X-rays and neutrons can be used to characterize the three-dimensional shape and internal structure of fossil material [40], but to date the former has been used far more extensively. X-ray computed tomography (X-ray CT) has been used by vertebrate palaeontologists for over 30 years (e.g. [20,21,41–43]). As high powered computers have become increasingly accessible, it has become a widely used and familiar approach for investigating hidden anatomical details (e.g. [44–46]), facilitating shape quantification (e.g. [47]), rendering vacuities (e.g. [48,49]), generating composite computer reconstructions [48,50], building biomechanical models (e.g. [51]) and isolating the enamel and dentine components of fossil teeth [24–26,52–55]. Neutron tomography has also been available for many years [56,57] and successfully used on plant fossils [58,59], but has only rarely been used for vertebrate fossil material and, rarer still, for quantitative analyses [60–67]. Given that X-rays and neutrons show different degrees of attenuation per chemical element ([60]; electronic supplementary material, figure S1), the two methods will likely provide different results for the same sample [58]. X-ray attenuation generally increases with atomic number and essentially measures density. The relationship between neutron attenuation coefficients and atomic number has no simple theoretical model, and neutrons can pass through many dense elements relatively easily [58,66]. Therefore, as very recently shown in some fossil primate teeth [68,69], it is possible that neutron tomography may be more informative than X-rays for fossil teeth where the different dental tissues have mineralized with similar density.

Here, we study a rare unworn dentary tooth of the eilenodontine *Eilenodon* (Upper Jurassic of North America) to better understand the dentition of eilenodontines and compare the potential of X-ray and neutron CT for measuring enamel thickness in fossil reptiles.

## 2. Material and methods

### 2.1. Materials

**Figure 1.**
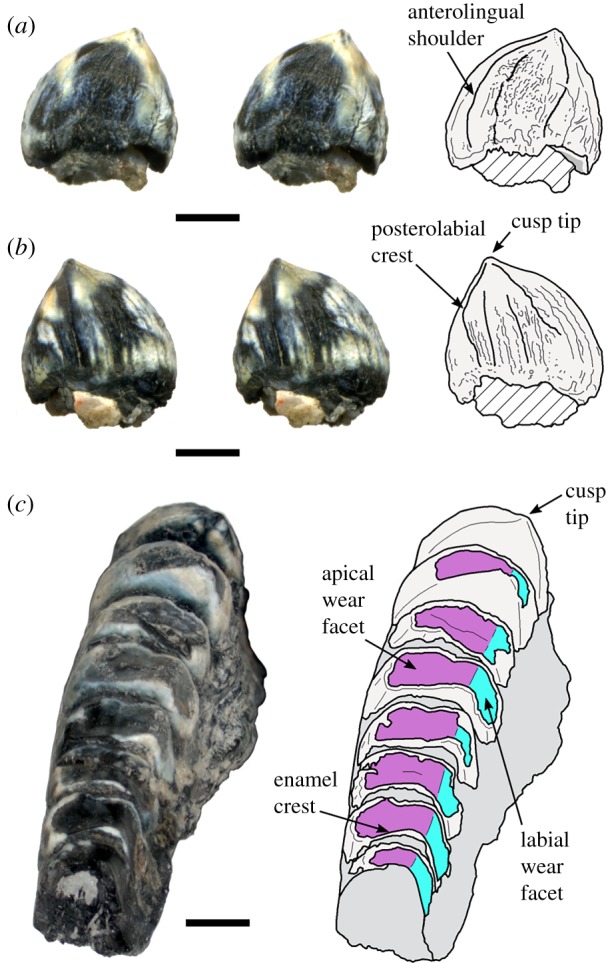
The left posteriormost tooth of *Eilenodon* (DMNH EPV.10685). (*a*) Anterior and (*b*) posterior stereopairs of the unworn isolated dentary tooth. (*c*) Anterodorsal view of a partial left dentary showing the apical and labial wear facets. Scale bar = 2 mm.

The dentary tooth is part of the material referred to *Eilenodon robustus* by Foster ([9]; DMNH EPV.10685), but it was not itself figured or specifically described. The material mainly comprises partial jaws and derives from Green Acres, eastern part of Garden Park, Colorado, USA, which exposes part of the Upper Jurassic Morrison Formation. The material was mainly collected from the surface (Bryan Small 2002, personal communication) and seems to represent a single adult individual. The tooth itself is relatively large for a lepidosaur: 4.7 mm labiolingual width, 3.3 mm mesiodistal length and approximately 4.2 mm apicobasal height (coronal height) (figure 1). The outer enamel surface of the tooth is also relatively complex for a lepidosaur, being bulbous and labiolingually wide with numerous conspicuous apicobasal ridges around the base. The cusp tip is pinched, laterally inclined, and gives rise to four subtle crests that run towards each corner of a dumbbell-shaped tooth base (a phenotype described as ‘crosslophed’ in [8]). The anterolingual crest is particularly prominent and forms a shoulder that contributes to a concave anterior surface. It is equivalent to the ‘medial crest’ of *Toxolophosaurus* [7] and probably also to the ‘shoulder’ [70], ‘medial flange’ [35] or ‘anteromedial flange’ [14] of other rhynchocephalians such as *Sphenodon*, and *Cynosphenodon* from the Early Jurassic of Mexico [35], and *Opisthias* also from the Upper Jurassic of USA [7]. The ventral edge of the anterolabial corner is damaged and there is a crack that extends internally from this.

The *Eilenodon* tooth specimen's shape, size and absence of wear compared to the other available dentition (figure 1*c*) suggest that it is almost certainly the posteriormost tooth from the left tooth row. Therefore, given how the dentition of rhynchocephalians is assembled, it is the youngest tooth in the tooth row (e.g. [70]). In more anteriorly placed teeth, a single continuous wear facet would be present on the apical and labial surface due to abrasion from the palatine and maxillary teeth, respectively [7,8].

Comparative material mainly comprised an adult *Sphenodon* (SAMA 70524) with relatively unworn posterior dentary teeth.

### 2.2. X-ray and neutron computed tomography

The specimen was X-ray scanned at Adelaide Microscopy using a Skyscan 1072 (Bruker, Billerica, MA, USA). It was held in place using a specially cut piece of polystyrene and scanned using the following parameters: 100 kV; 80 µA; 0.5 mm focal spot; 1601 projections and a pixel size of 6.5 µm (0.065 mm). To reduce the effects of beam hardening, the X-rays were filtered with a 1.0 mm thick aluminium plate. Tomographic reconstruction of the raw data was performed in Bruker NRecon v.1.6.10.2 using a beam hardening correction 100%, ring artefact correction 20 with a dynamic range of 0.015–0.150, producing 801 16-bit TIFF slices.

The specimen was neutron scanned using DINGO, the Radiography/Tomography/Imaging Station at the Australian Centre for Neutron Scattering, Australian Nuclear Science and Technology Organisation, Sydney. The DINGO instrument uses a quasi-parallel collimated beam of thermal neutrons generated by the OPAL research reactor. The specimen was scanned with a collimation ratio (*L*/*D*) of 1000 [71,72] to ensure the highest available spatial resolution, where *L* is the neutron aperture-to-detector length and *D* is the neutron aperture diameter. The specimen was wrapped in aluminium foil and inserted into a purposely prepared aluminium holder. The field of view was set to 50 × 50 mm^2^ and scan time was 36 h with spatial resolution of 26 µm (0.026 mm). Neutrons were converted to photons using a ^6^LiF/ZnS(Ag) scintillator; photons were then detected by an Andor IKON-L CCD camera (liquid cooled, 16-bit, 2048 × 2048 pixels) coupled with a Makro Planar 100 mm Carl Zeiss lens. A total of 1440 projections with an exposure length of 90 s were obtained every 0.25° as the sample was rotated 360° about its vertical axis with an exposure length of 90 s. Tomographic reconstruction of the raw data was performed using Octopus Reconstruction v.8.8 (Inside Matters NV). Slices were kept at 16-bit. The dataset was despeckled to smooth the image by replacing aberrant values by the mean value of their neighbours. An anisotropic diffusion filter was applied to further reduce noise while enhancing edge-contrast. To determine the value of a voxel, the algorithm compares the value of that voxel with the value of its six neighbours. If the difference does not exceed the diffusion stop criterion (3327), diffusion is applied. The algorithm was iterated five times.

**Figure 2.**
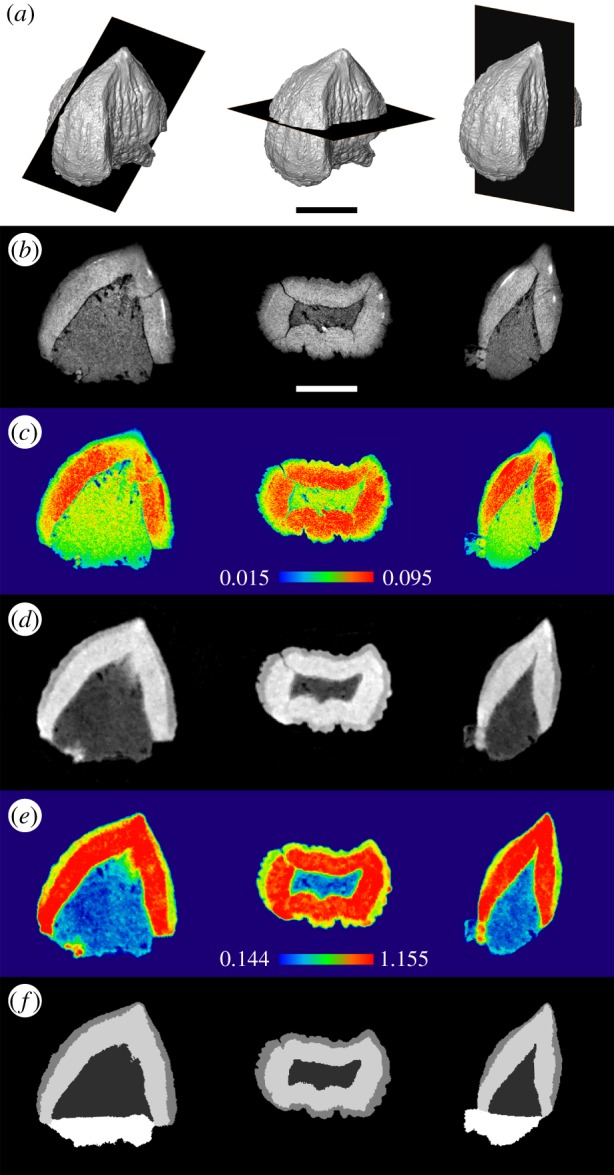
Sections of the left posteriormost tooth of *Eilenodon* (DMNH EPV.10685) made using X-ray CT and neutron tomography. (*a*) Computer models showing the location of the coronal, horizontal and mesiodistal section. (*b*) Final segmented model based on neutron attenuation. (*c*) X-ray results. (*d*) Neutron results. (*e*) X-ray results coloured according to attenuation. (*f*) Neutron results artificially coloured according to attenuation. Scale bar = 2 mm.

Both datasets were examined using avizo 8.01 (Visualisation Science Group, SAS). In avizo, the datasets were aligned so that precisely comparable sections could be compared (figure 2). Computer models of the outer external surface of the tooth were made using both datasets. Both datasets showed three internal tooth components consistent with enamel, dentine and pulp. However, the enamel–dentine junction was only distinct enough for meaningful segmentation in the neutron dataset. As is typical for fossil specimens, this segmentation was achieved using a combination of thresholding and manual editing (e.g. [73]). First, a threshold was applied to capture the entire tooth that successfully represented the appearance of the outer external surface. Second, a threshold was applied to isolate the majority of outer voxels (enamel). Third, thresholding was used to delimit the boundary between the middle layer (dentine) and the inner cavity (pulp). Fourth, each layer was visually inspected and isolated voxels were added to the surrounding material. The ventral margin of the enamel was also extended to include some voxels that were particularly dense relative to more obvious dentine. Fifth, the base of the pulp cavity was defined with a near horizontal line between the ventralmost preserved enamel and dentine (electronic supplementary material, protocol).

Enamel and dentine thickness were measured using the avizo thickness module which measures the distance between opposing triangles in the mesh of an unsmoothed (existing weights) surface file of the neutron dataset segmentations. This approach provides a histogram of surface element number by underlying enamel thickness.

The *Sphenodon* (SAMA 70524) specimen was scanned with the following parameters: 100 kV; 400 µA; 1199 X-ray projections and a pixel size of 25 µm (0.025 mm). A molybdenum target was used with a 0.5 mm Al filter to maximize contrast in the specimen. Volume reconstruction of the micro-CT data was performed using the phoenix datos|x reconstruction software (GE Sensing & Inspection Technologies) and data were exported as 32-bit float volume files. Computer models of the outer enamel surface were made in avizo using thresholding to measure tooth shape. Although the outermost edges of the teeth comprised denser material that likely represents enamel, the boundary between enamel and dentine is not very clear and precludes adequate segmentation.

### 2.3. Force resistance

Teeth are essentially composed of a hard but brittle shell (enamel) and a tough but deformable interior (dentine) capable of sustaining frequent loading [29]. The two main possibilities for crown fracture depend on whether the load is (i) concentrated over a small contact area, in which case the fracture load estimate assumes that the enamel will flex on the underlying dentine, so producing a radial crack that runs from inside-out, or (ii) spreads over a large area of the crown, in which case the failure zone is likely to start low down on the crown around the margin of the base. The resistance of the crown to fracture is dependent on tooth size (*R*, the tooth radius at the crown base), the thickness of the enamel *t*, its toughness *K*_c_ and a dimensionless coefficient *c* related to tooth shape. The peak force at fracture is [29]2.1
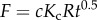


**Figure 3.**
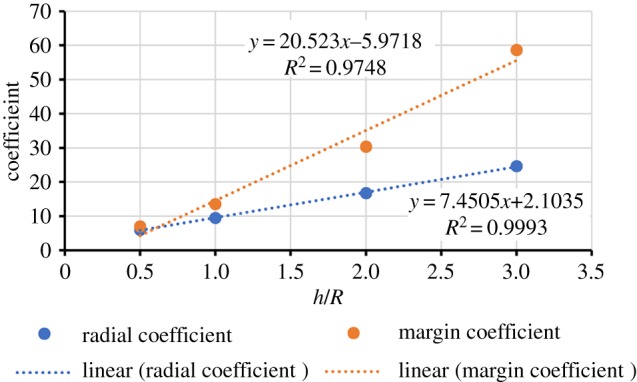
The relationship between coefficient *c* and tooth shape with respect to tooth height divided by radius. The experimental results in Barani *et al*. [74] as given in their fig. 5 yield the following plot for the increase in the coefficient with change in *h*/*R*. A bunodont tooth is given an *h*/*R* of 0.5. For *h*/*R* > 3.0, the coefficients would begin to plateau. For unworn posterior teeth, *Sphenodon* has an *h*/*R* value of between 1.95 and 2.32, whereas *Eilenodon* has an *h*/*R* close to 2. (Online version in colour.)

The value of the coefficient *c* depends on tooth shape: for low-crowned teeth, it is 6–8 [29], depending where fractures initiate, but it rises with crown height to reach 50–55 for pencil-like or hypsodont teeth [74]. An estimate can be made for adult *Sphenodon* using the dimensions of three unworn posterior dentary teeth (electronic supplementary material, table S1): a mean radius (*R*) of approximately 0.88–0.98 mm (electronic supplementary material, table S1) and apicobasal height (*h*) of 1.7–2.2 mm (electronic supplementary material, table S1) gives a *h*/*R* between 1.95 and 2.32 (electronic supplementary material, table S1) and therefore a *c* of between 17 and 42 (figure 3; electronic supplementary material, table S2). Enamel thickness is approximately 0.11 mm (range 0.08–0.14 mm) [36,37], while *K*_c_ is 0.21–0.32 MPa m^0.5^ [38]. Equation (2.1) predicts a maximum sustainable force of one dentary tooth to be 33–143 N (electronic supplementary material, table S3). This range of values is broad but also explicit. The teeth have half the resistance to radial cracks than they do to marginal cracks (regardless of toughness), and the higher value for toughness is associated with an approximately 50% greater estimated force resistance. Estimates based on enamel thickness of 0.11 and 0.13 mm differ by approximately 10%, whereas variation in estimates among the three teeth due to differences in tooth shape is consistently less than 25% (when toughness and crack type are kept constant).

Estimates of critical loading for teeth may permit estimates of bite force. For wild adult *Sphenodon*, the maximum bite force measured at the front of the mouth is 175–275 N [75]. Bite forces may be twice as great at the posterior end of the tooth row due to lever mechanics [76]: approximately 550 N. Therefore, the maximum possible bite forces are much greater than the critical failure for one dentary tooth. However, such forces would very likely be shared across multiple teeth due to the shape of the jaws, arrangement of tooth rows and because the greatest loading may not be applied until the jaws are fully engaged [14,77]. Moreover, some specimens of adult jaws of *Sphenodon* do exhibit broken tooth crowns which probably represent instances where loading from a particularly forceful bite was concentrated on an unusually small number of teeth. Available measurements suggest that the greatest anterior bite forces possible (275 N) are 1.9 and 8.4 times greater than the highest and lowest critical load estimate for the posterior dentary teeth, respectively.

To estimate critical loading for the unworn dentary tooth of *Eilenodon*, we use the same enamel toughness as reported for *Sphenodon* [38], but measure the enamel radius, and enamel thickness from the surface models of the unworn tooth of *Eilenodon* generated using neutron CT. To provide a very general estimate for the anterior bite force of *Eilenodon*, we apply the relationship found in *Sphenodon* between anterior bite force in *Sphenodon* and critical loading of a posterior dentary tooth.

## 3. Results

### 3.1. Comparison of scanning methods

Both the X-rays and neutrons are successful at representing the outer enamel surface. The X-ray dataset reveals more detail due to the greater resolution; nonetheless, all major features are visible in the neutron model (e.g. the anterolingual shoulder, the apicobasal ridges, the acuminate cusp tip and the posterior ridge). The monochrome models make it easier to appreciate the surface detail than photographs of the fossil which is a mottled black, white and blue.

**Figure 4.**
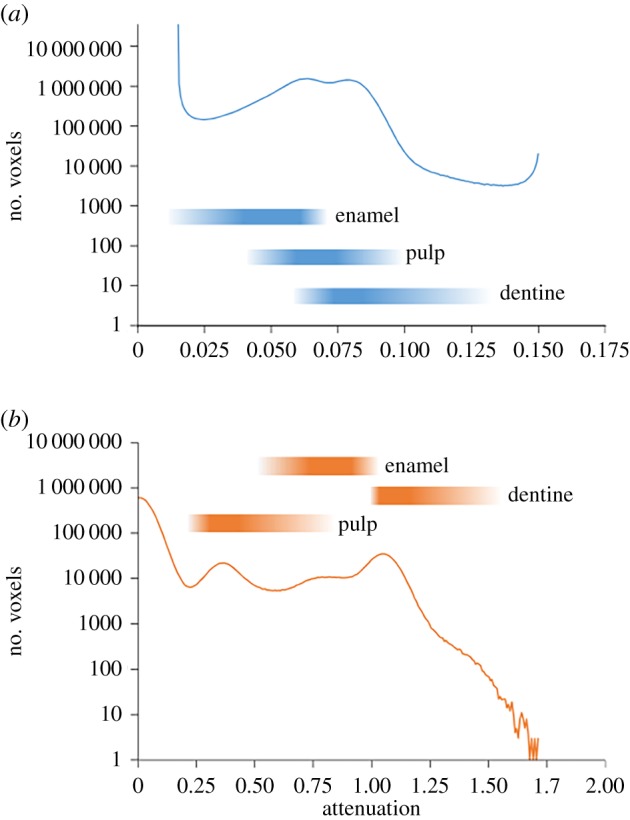
Attenuation plotted against the number of voxels in the dataset representing that attenuation according to (*a*) X-rays and (*b*) neutrons. The coloured blocks indicate the typical attenuation values of particular dental components in the fossil specimen. Attenuation values for the different dental tissues are relatively distinct in the neutron dataset but not that of the X-ray dataset. (Online version in colour.)

The X-ray dataset is inadequate for interpretation of the enamel dentine junction (figure 2*b,c*). In the attenuation distribution, there is a broad peak between 0.025 and 0.10 which encompasses most of the voxels corresponding to tooth tissues (figures 2*c* and 4*a*, and table 1; electronic supplementary material, table S4): enamel (0.0191 to approximately 0.0791 with most values above 0.0493), pulp (0.0551 and 0.0837) and dentine (approx. 0.0722 or greater). The boundary between enamel and dentine is rarely distinct, and in most regions, it is not possible to tell where one material ends and the other begins (figure 2*b*,*c*). There are also some clusters of voxels with values greater than 0.01066 which appear to lie within the dentine against the boundary with the enamel. However, they are not continuous enough to permit meaningful separation of the two components.

**Table 1.** Attenuation values that typically represent particular tooth components.

material	X-ray dataset	neutron dataset
enamel	0.0191 to approximately 0.0791	0.5199–1.0399
dentine	0.0722 or more	1.0254–1.5887
pulp	0.0551–0.0837	0.2311–0.9243

Neutrons are more effective than X-rays at revealing the internal structure of the tooth despite the neutrons having lower resolution measurement (figure 2*d*,*e*). In the attenuation distribution of the neutron dataset, there are distinct peaks at 0.35 and 1.15, as well as a more subtle peak at 0.76 (figures 2*e* and 4*b*, and table 1). The majority of the enamel has an attenuation of between 0.72 and 1.01, but also includes voxels with a wider range of attenuations (0.51–1.04). There are also two regions of material (one in the posterolabial corner and the other at the base of the anterolingual shoulder) with very high attenuation values (1.30–1.70) that are interpreted to be part of the enamel. Attenuation within the dentine is generally between 1.04 and 1.30, but there are also a few regions where it approaches 1.70. The boundary used to delimit adjacent enamel and dentine during segmentation was 1.0399. The material in the pulp cavity is typically between 0.23 and 0.58, but near the boundary with the dentine it is close to 0.87 and the boundary itself inferred during segmentation was 0.9185.

### 3.2. Tooth anatomy

**Figure 5.**
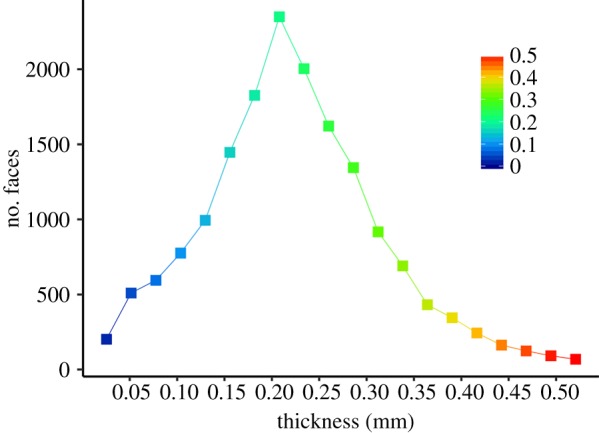
Enamel thickness frequency according to the surface element number of an unsmoothed surface model of the enamel as segmentation of the neutron CT dataset. The colour gradient is the same as used for figure 6*d*. The data are binned at intervals of 0.026 mm which corresponds to the isometric voxel dimensions.

**Figure 6.**
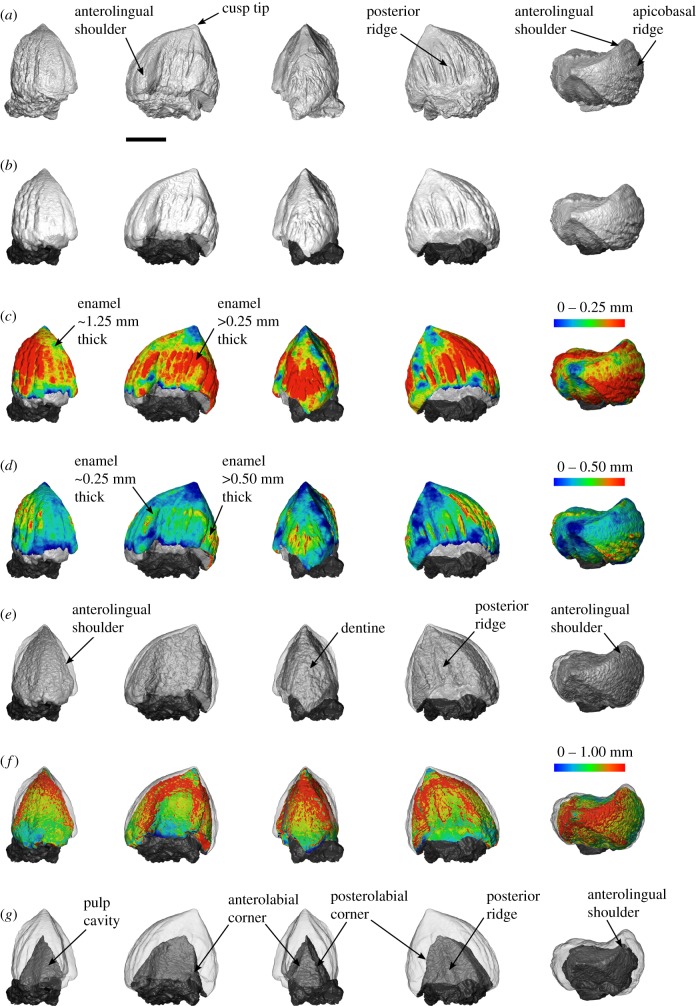
Computer models of the left posterior most tooth of *Eilenodon* (DMNH EPV.10685) built using (*a*) X-ray and (*b*–*g*) neutron CT attenuation data shown in lingual, mesial, labial, distal and apical view. (*a*–*c*) Outer enamel surface (OES). (*c*,*d*) OES colour coded for enamel thickness with the thickest enamel in red and thinnest in blue. (*e*) The enamel–dentine junction as shown with a transparent OES and opaque dentine in grey. (*f*) The dentine colour coded for enamel thickness with the thickest dentine in red and thinnest in blue. (*g*) The shape of the volume representing the pulp cavity opaque with a transparent OES. Scale bar = 2 mm.

The neutron dataset shows that enamel is a major component of the tooth. For the portion of tooth preserved, the volume of enamel is 6.60 mm^3^, dentine 14.15 mm^3^ and pulp cavity 5.54 mm^3^. Therefore, the enamel volume is nearly equal to half that of the dentine (47%). Based on neutron data, the modal enamel thickness in *Eilenodon* is approximately 0.20 mm thick (figure 5), but the thickness is surprisingly uneven and generally between 0.15 and 0.30 mm (figure 6). The enamel is thinnest at the cusp tip (less than 0.10 mm) and thickest at the apicobasal ridges (0.30–0.50 mm) (figure 6*c*,*d*). The apicobasal ridges present on the lingual and posterior surface of the tooth are not present at the enamel dentine junction and, therefore, represent thickened enamel (figures 2*d*,*e* and 6*c*). The dentine without the enamel (figure 6*e*) bears a close similarity to the overall tooth shape (figure 6*e*,*f*): there is an obvious anterolingual shoulder, an anterolabial corner (although obscured by damage), a clear posterolingual corner and a posterolabial corner. In addition, there is an apicobasal ridge running along the posterior midline. The dentine is generally between 0.40 and 0.80 mm thick with the thickest portions being along the corner ridges and posterior ridge. A segmentation of the pulp cavity reveals a pyramid-like structure with four crests running to each corner, an expanded anterolingual shoulder as well as a posterior apicobasal ridge (figure 6*g*).

### 3.3. Force resistance

Using the measurable properties of the fossil tooth (enamel thickness, size and shape), we estimate that the dentary teeth of *Eilenodon* had 2.3–3.1 times the resistance to fracture than those of the modern *Sphenodon*. For *Eilenodon*, we know that the posterior teeth have a crown radius of approximately 0.002 mm, an unworn apicobasal height of 4.2 mm and, therefore, a *h*/*R* of between 1.9 and 2.1 (electronic supplementary material, table S5) and *c* of between 17 and 36. The *h*/*R* of *Eilenodon* and *Sphenodon* is similar because although the teeth of *Eilenodon* are relatively wider labiolingually, they are also relatively short mesiodistally and the proportional unworn apicobasal height is similar. If we use the modal thickness of 0.20 mm and assume that the teeth of *Eilenodon* have the same enamel toughness as *Sphenodon* [38], we obtain a critical load estimate of 101–325 N per tooth (electronic supplementary material, table S5); it was 33–143 N in *Sphenodon*. As in *Sphenodon*, the load would be spread across multiple teeth. Assuming that the relationship between anterior bite force and the resistance to fracture for an individual unworn dentary tooth present in *Sphenodon* is the same for *Eilenodon* (1.9 and 8.4 times greater than the highest and lowest critical load estimate for the dentary teeth, respectively), we would predict a maximum anterior bite force of 625–843 N for *Eilenodon*. This estimate is much greater than *Sphenodon* (275 N) but not entirely unreasonable, given that *Eilenodon* is much larger than *Sphenodon* (maximum skull length = 110 versus 70 mm) [2,13]. If *Eilenodon* possessed the same relationship between skull length and bite force as *Sphenodon* [75], animals with a skull length of 110 mm would have an anterior bite force close to 500 N. Lizards with skull lengths of approximately 100 mm (*Salvator merianae* and *Dracaena guianensis*) are reported to have anterior bite forces of approximately 500 N [78]. It is possible that the maximum bite force of the largest individuals of *D*. *guianensis* are underestimated given the relationships shown in figures 1 and 3 in Schaerlaeken *et al*. ([78], fig. 3). Overall, this result highlights the need for wider surveys of bite force among living lepidosaurs and a greater understanding how bite force relates to tooth structure.

## 4. Discussion

Once again, examination of a fossil member of Rhynchocephalia highlights the diversity of this group and demonstrates that *Sphenodon* is not necessarily representative of its Mesozoic relatives [2,12]. The greater enamel thickness in *Eilenodon*, compared with the smaller *Sphenodon* (and smaller still, *Sphenocondor*), may be related to scaling but examination of additional Rhynchocephalia, such as *Clevosaurus* from the Triassic of the UK [18], are required to test this hypothesis. Nevertheless, the tooth enamel thickness of *Eilenodon* appears to be greater than that of crocodylians [31,32] and some dinosaurs [33]. Wider surveys of other reptiles including unusual taxa such as herbivorous crocodylians [79] and aquatic placodonts [46] are necessary to fully appreciate the macroevolution of enamel thickness in amniotes. The relationship between enamel thickness and enamel microstructure [80] also requires further investigation.

The differences in enamel thickness between *Eilenodon* and *Sphenodon* likely reflect different functional demands. Compared to *Sphenodon*, and in combination with the greater tooth size, the thicker enamel around the base of the tooth in *Eilenodon* would have been able to sustain up to three times the load before failure due to a marginal crack. Also, as previous authors have discussed, the thickened enamel also permits the establishment of long-lasting wear facets that have sharp edges for shredding tough material [2,3,7,8]. The relatively thin enamel at the tooth cusp tip in *Eilenodon* may be necessary for enabling the functional wear facets to be acquired as early as possible.

The apicobasal ridges on the outer surface of teeth are not unique to *Eilenodon* but can be found in many vertebrate taxa (e.g. [81–83]), including other Rhynchocephalia (e.g. [84], but not *Sphenodon* [35]). The ridges would provide enhanced tooth penetration [81,82], greater grip [83,85] and additional abrasive edges for reducing food items [82], and they may have helped transfer stresses from the cusp tip [83,86]. The fluting between the ridges would help remove fluids and food fragments from the cutting surfaces of the teeth [82,87].

*Eilenodon* would have likely fed on a range of herbaceous to arbustive plants, and perhaps opportunistically the odd insect, but a potentially preferred food source known from fossil remains in the Morrison Formation was likely to be *Equisetum* (horsetails). Extant members of this genus are rich in energy, protein and phosphorous, and are easy to digest [88,89]. However, even in the earliest forms [90], extensive silica deposition in the outer tissues of the stems serves to stiffen them, making it potentially abrasive [91]. Deep to this outer layer is a region of softer tissue with high toughness encountered around the vascular bundles. Subdivision of the stem requires bladed teeth, which *Eilenodon* seems to have acquired via wear facet formation. However, by the time this vascular region is loaded, the stem tissue will have spread itself over the crown. When contacting the outer crust, the enamel will tend to flex on the underlying dentine below the point of contact, potentially producing dangerous radial cracks running out from the enamel–dentine junction towards the tooth surface. As the vascular bundles are cut, any potential cuspal fractures would be suppressed by the highly compressive stress field produced by a smothering food contact. Failure zone is then likely to start low down the crown in the enamel around the margins of the crown base [29].

The thicker enamel would also prolong crown life by increasing resistance to enamel wear. This depends on the relative hardness of abrasive (plant silica in this case) and enamel, the angle of contact and on the toughness of enamel. Phytoliths in extant plants have an upper hardness limit of 2–3 GPa [92–95], while the hardness of mammalian enamel ranges from 3 to 6 GPa, increasing towards the tooth surface [96]. This makes plant silica a rubbing agent on mammalian enamel, only indirectly capable of removing enamel tissue from multiple contacts on a plastically deformed enamel surface. In prismless reptilian enamels, however, hardness is lower, typically being approximately 3–4 GPa [32,37,97], which reflects its lower mineral content [32,38,98]. Furthermore, the toughness of *Sphenodon* enamel is much lower than that of mammals [99], also making plant silica much more of an abrasive threat.

**Figure 7.**
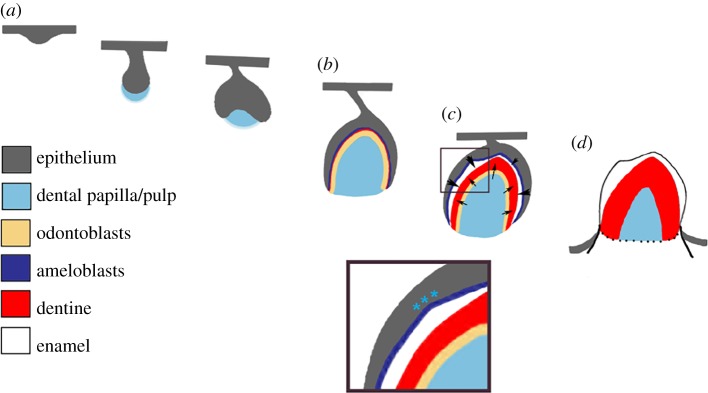
Hypothesis of tooth development in *Eilenodon* following enamel thickness and tooth development in other amniotes. (*a*) Early stages of tooth development common across all toothed vertebrates (thickening, bud and cap). (*b*) Bell stage with formation of odontoblasts (yellow band) from the dental papilla and ameloblasts (dark blue band) from the adjacent inner enamel epithelium. A small amount of dentine (red) is laid down by the odontoblasts at this stage. (*c*) Late bell stage. More dentine (red) is laid down by the odontoblasts (arrows) in a uniform pattern mimicking the shape of the papilla. By contrast, the ameloblasts in *Eilenodon* lay down enamel (white) in a more irregular pattern (indicated by large and smaller arrows) producing a tooth with varied thickness of enamel depending on location. (*d*) Final erupted tooth. The odontoblasts continue to produce dentine, but the ameloblast layer is lost as the tooth erupts exposing the enamel to the surface. Asterisks indicate areas of heightened ameloblast activity.

The internal structure of the tooth can also allow us to hypothesize how the tooth formed given what we know about tooth development in extant taxa (e.g. [28,30,100,101]). The major features of the outer external surface that are also visible at the enamel–dentine junction and pulp cavity are likely due to folding of the epithelium: the cusp tip, four crests, four corners, anterior concavity and posterior ridge. However, the bulbous nature of the teeth associated with uneven enamel thickness and apicobasal ridges around the base of the tooth are due to uneven enamel deposition by ameloblasts (figure 7). Uneven deposition is how the bicuspid teeth develop in skinks [102] and geckos [30,100,101]. Despite the labially inclined cusp tip and wider labiolingual dimension [3], the pyramid-like shape of the dentine and pulp cavity of *Eilenodon* resembles the outer surface shape of the posterior dentary teeth of phylogenetically nested rhynchocephalians that have thin enamel [35], including *Sphenodon*, *Cynosphenodon* and, in particular, *Opisthias*. Therefore, the major differences between eilenodontines and these other rhynchocephalians are likely due to differences in ameloblast activity rather than initial folding of the epithelium prior to differentiation.

We have shown that in at least some instances (see also [69]), neutron tomography may provide favourable contrast compared to X-ray tomography and, therefore, warrants greater use among palaeontologists than practiced to date (e.g. [72,103]). Given the variation in resolution among different X-ray and neutron scanners, as well as in the composition of fossil specimens, our comparison of the two approaches is not comprehensive. The neutron tomography used here does not have the same resolution (and likely accuracy) as the X-ray tomography used elsewhere for primates (e.g. [52]). Nevertheless, the differences we found seem related to differences in attenuation within a specific specimen, and not resolution (cf. [69]). Other fossil material from the Morrison Formation may respond in a similar way to the specimen examined here, but the Formation is extensive (approx. 1.5 km^2^) and mineralisation may vary considerably among localities [104]. X-ray CT has been used successfully to isolate bone [44] and plant material [89,105] from the surrounding matrix of this rock unit. However, to our knowledge, examination of dental tissues in other specimens has not yet been attempted.

## 5. Conclusion

Neutron CT successfully allowed enamel and dentine to be differentiated within the fossil tooth despite the boundary being unclear in higher-resolution X-ray tomography. This example (along with [69]) highlights the potential of neutron tomography as a viable alternative to conventional X-ray tomography. We show that *Eilenodon* has enamel which is twice as thick as that of *Sphenodon*, but it is unevenly distributed. The thick enamel around the main body of the tooth would resist marginal cracks and, along with tooth size, facilitate critical loading two to three times greater than calculated for *Sphenodon*. The relatively thin enamel at the tooth cusp tip in *Eilenodon* may be necessary for enabling the long-lasting functional apical wear facets to be acquired as ontogenetically early as possible. The shape of the enamel dentine junction indicates that tooth development in *Eilenodon* and *Sphenodon* involved similar folding of the epithelium, but were different with respect to ameloblast activity.

## Supplementary Material

SI Segmentation Protocol

## Supplementary Material

SI Additional Figures

## Supplementary Material

SI Table 1

## Supplementary Material

SI Table 2

## Supplementary Material

SI Table 3

## Supplementary Material

SI Table 4

## Supplementary Material

SI Table 5

## Supplementary Material

3D animation tooth_3

## Data accessibility

The X-ray micro-CT dataset, neutron CT dataset and surface models are available on Morphosource (reptile enamel thickness P485).
