# Supplementary material for: Neutron scanning reveals unexpected complexity in the enamel thickness of an herbivorous Jurassic reptile
Source: J R Soc Interface. 2018 Jun 13;15(143):20180039. doi: 10.1098/rsif.2018.0039 (PMC6030635; doi:10.1098/rsif.2018.0039)
Supplement: SI Segmentation Protocol [file rsif20180039supp1.pdf]

SI - Segmentation protocol and additional notes

Neutron dataset slices

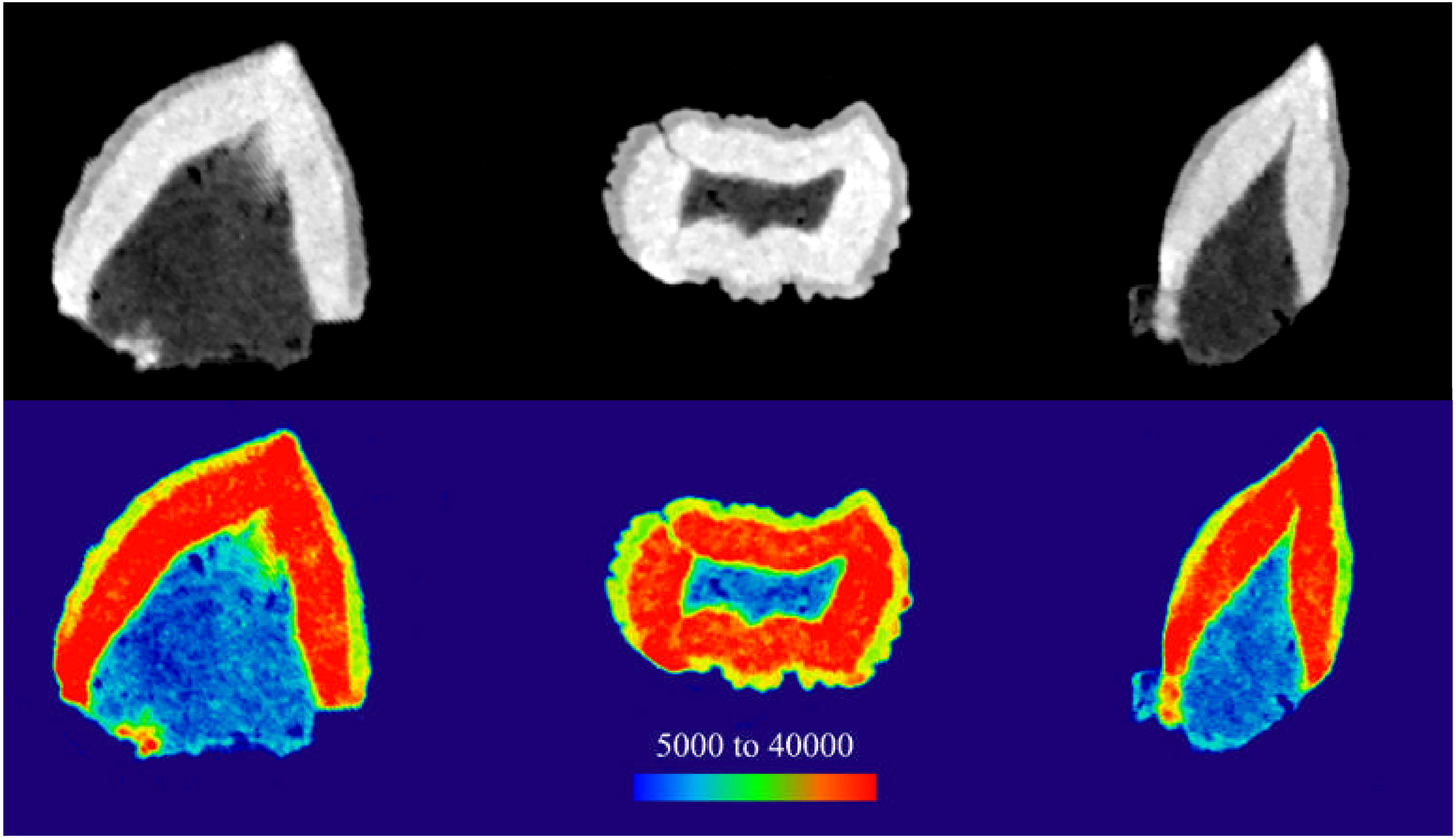

Neutron dataset slices

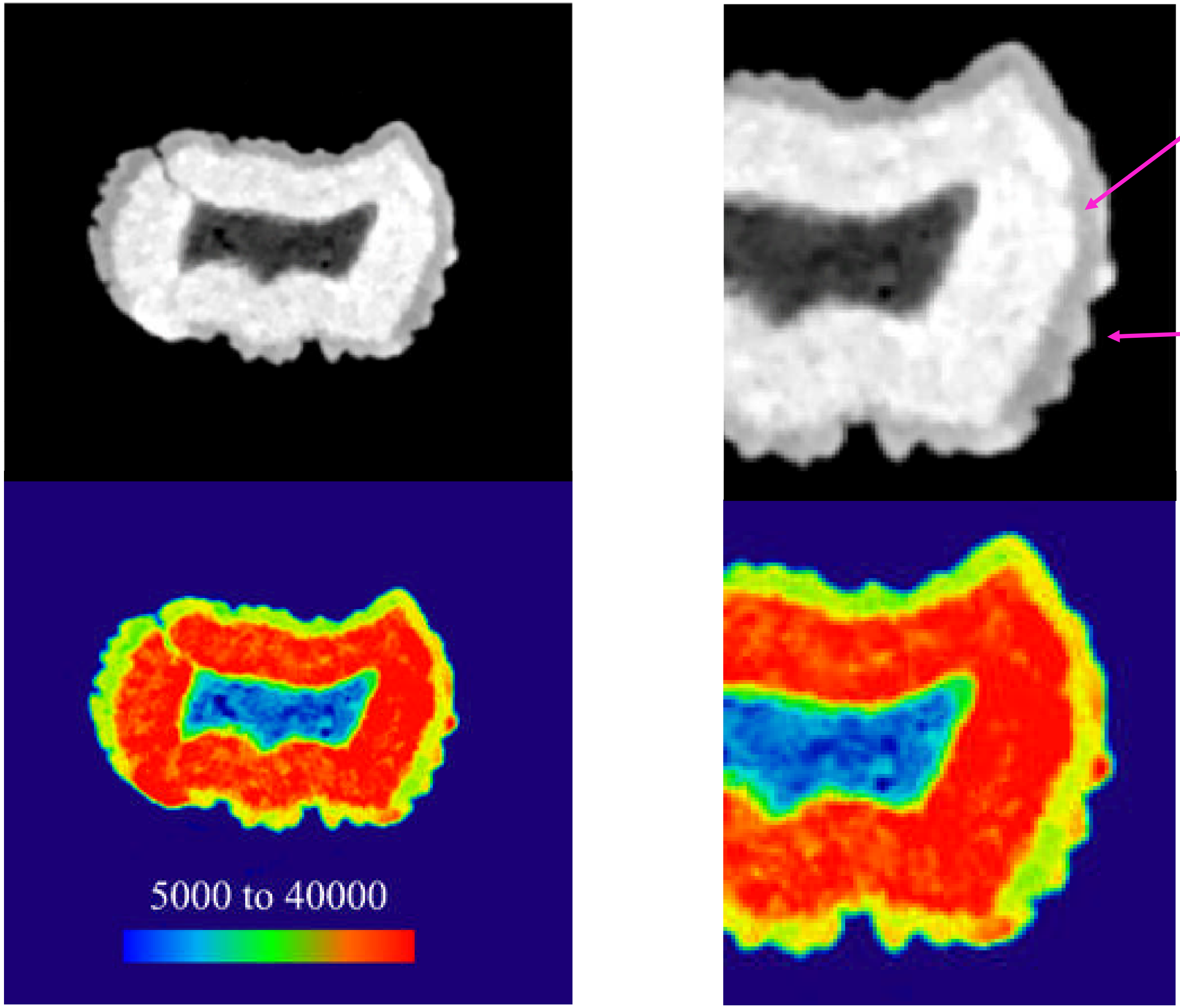

Outer layer thickness is consistent that of the enamel as it appears when exposed at wear facets

Ridges are not present at the enamel-dentine junction and therefore represent thickening of the enamel not folding.

The 16-bit grayscale values (as given by Avizo) that typically represent different tooth components within the neutron dataset. The greyscale values from the neutron dataset are proportional to raw attenuation by x 0.000028885.

Material	Neutrons
Enamel	18000 to 36000
Dentine	~35500 to 55000
Pulp	8000 to 32000

General segmentation protocol

1. Applied a threshold of 17750 to segment tooth, based on overall appearance.
2. Applied a threshold of 17750 for 36000 to isolate the main part of the enamel, based on overall appearance.
3. Lock enamel and exterior materials. Applied a threshold 31800 (to 55000) to define the boundary between the dentine and pulp cavity. Unlock enamel.
4. Manually segment to remove islands and make some parts continuous. Inspect slices in all three planes and manually fill vacuities within the dentine, pulp cavity, and bone.
5. Separate the bone and pulp cavity with a near horizontal line based on the available tooth anatomy

Example neutron slices

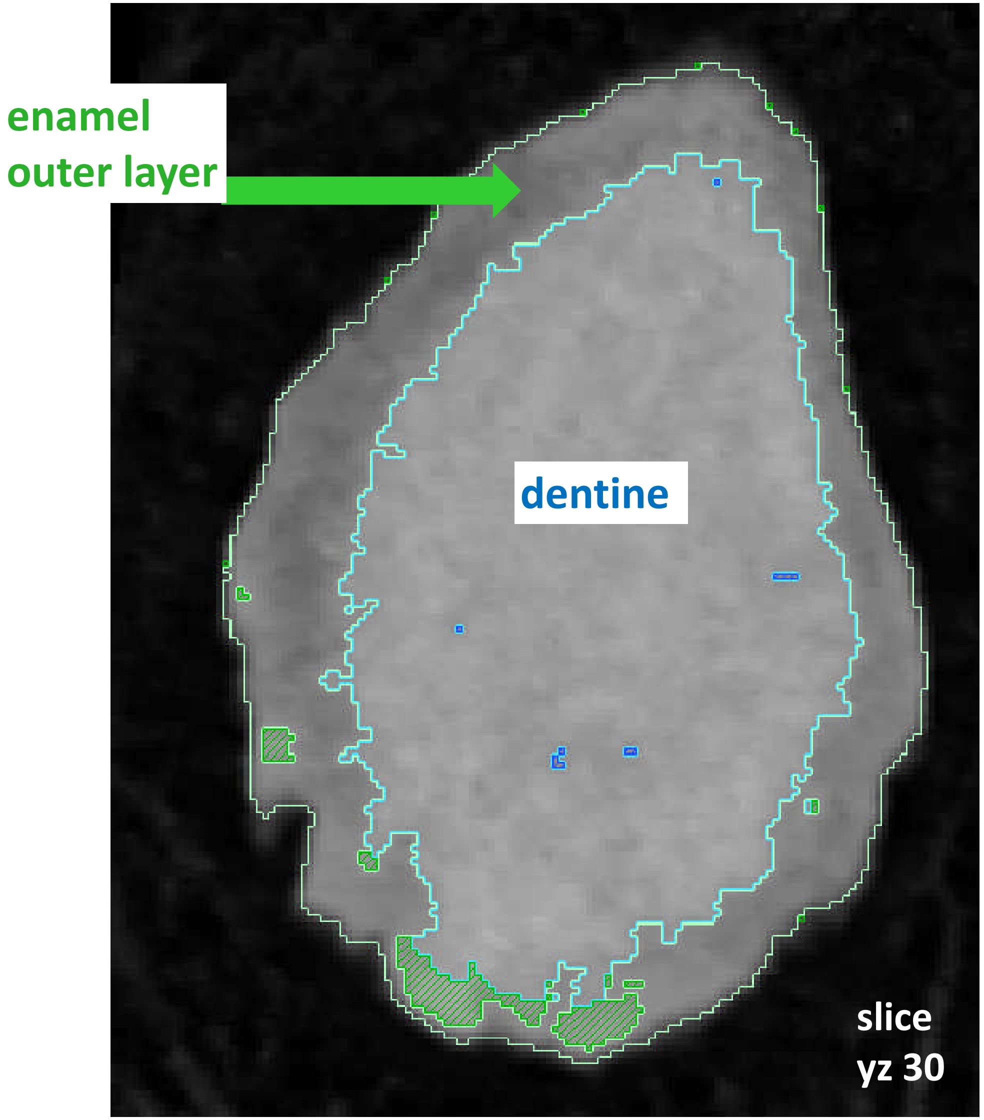

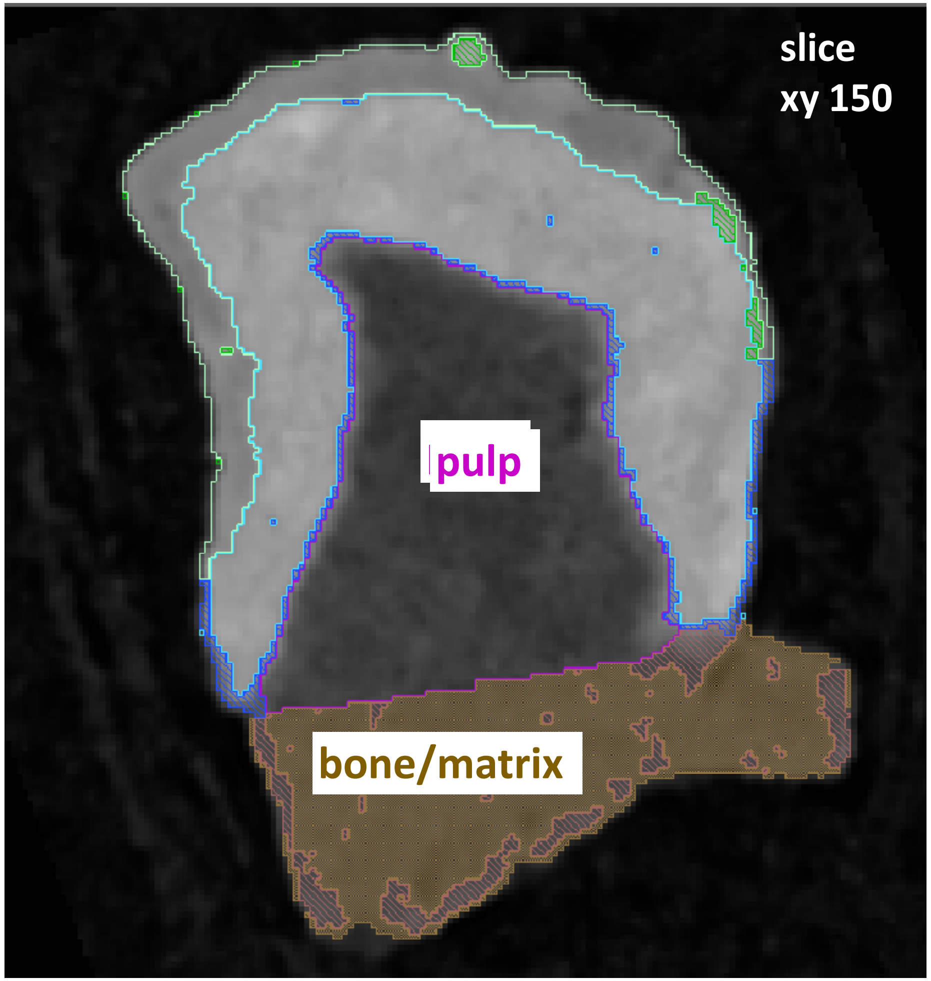

Numbers relate to the segmentation protocol

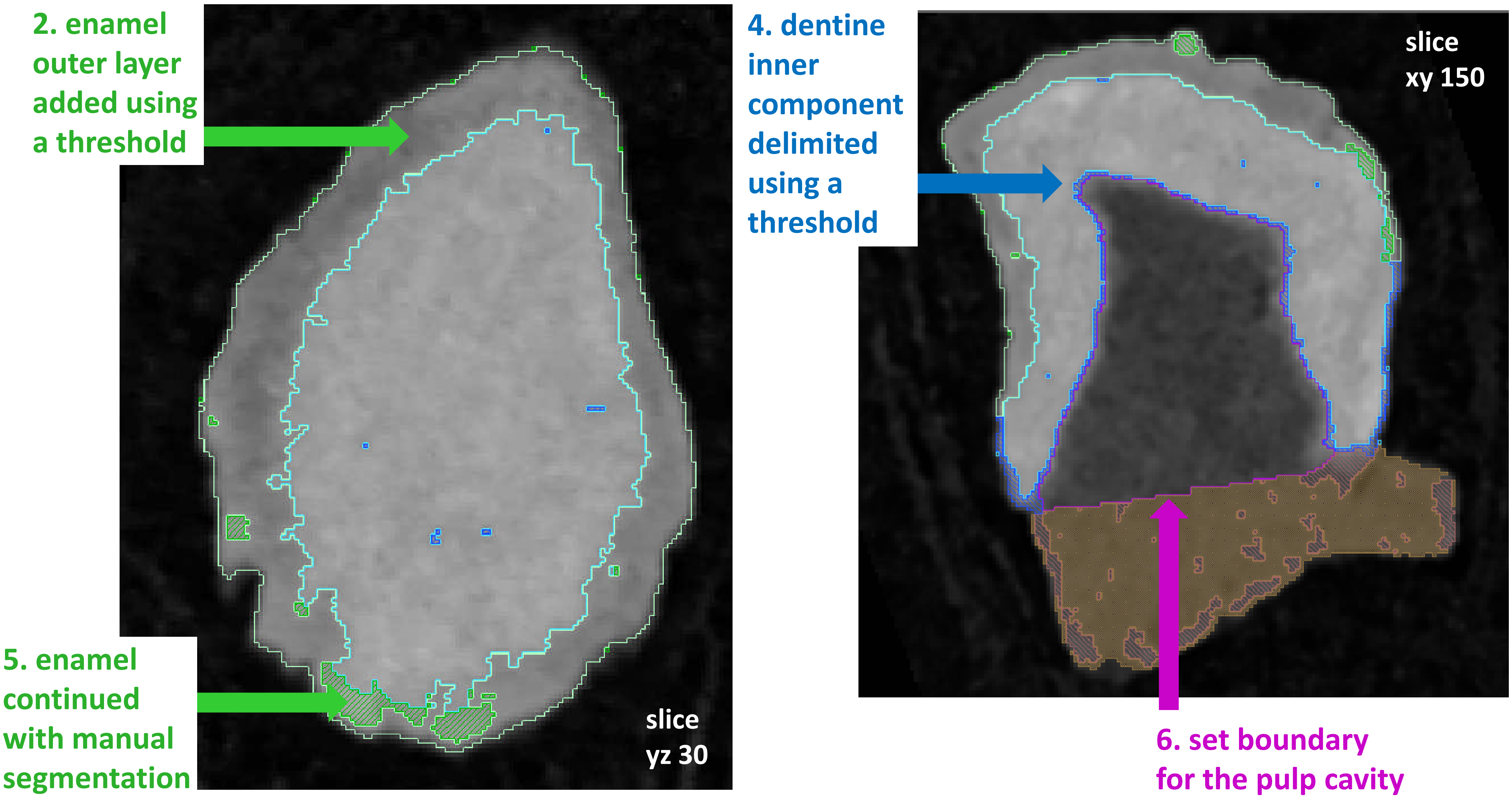

Numbers relate to the segmentation protocol

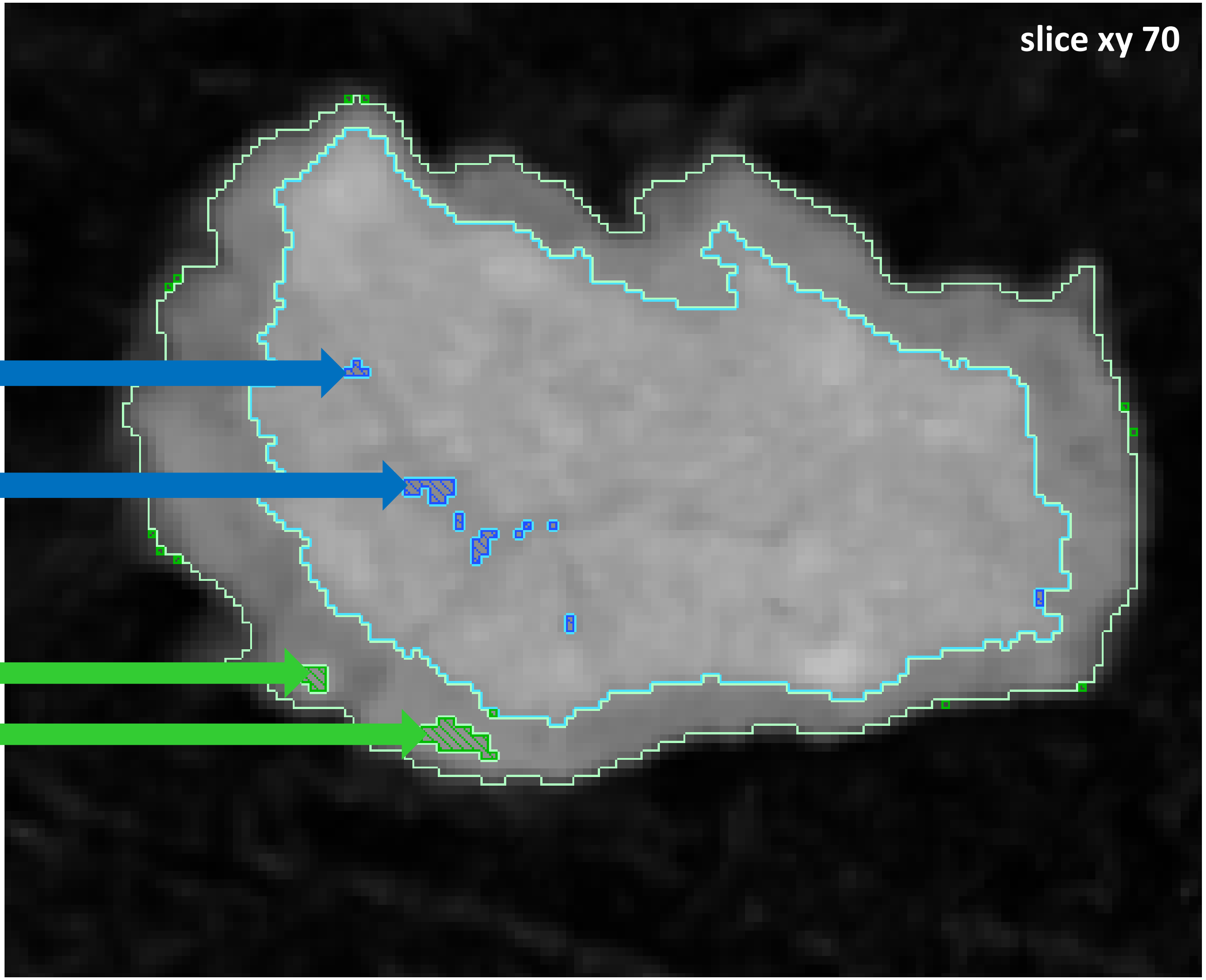

7. island added to the dentine using manual segmentation

7. island added to the enamel using manual segmentation

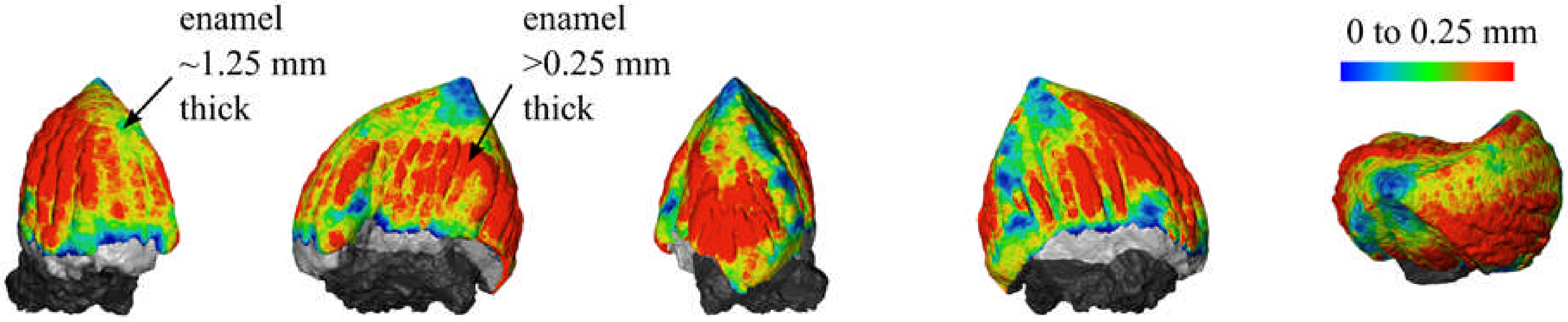

enamel
added
manually

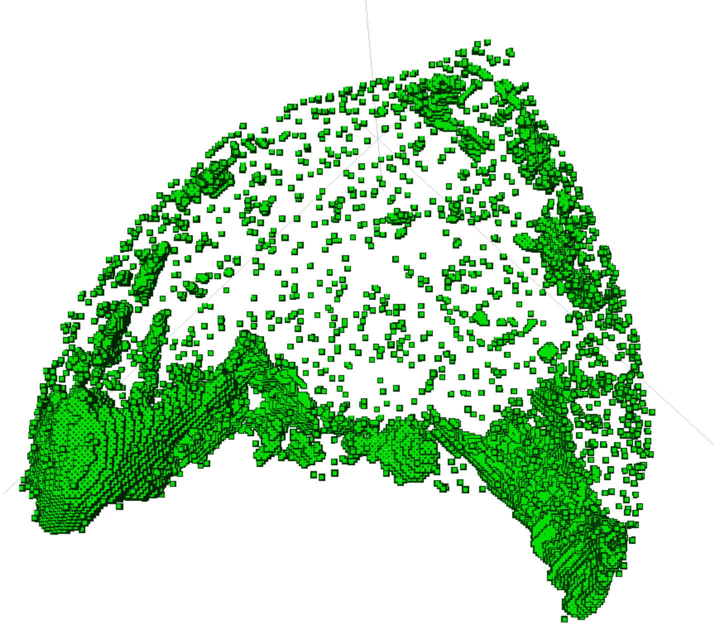

the enamel at the very base of the tooth required the most interpretation and manual segmentation not the ridges that show variation in thickness or the thickest part of the enamel.

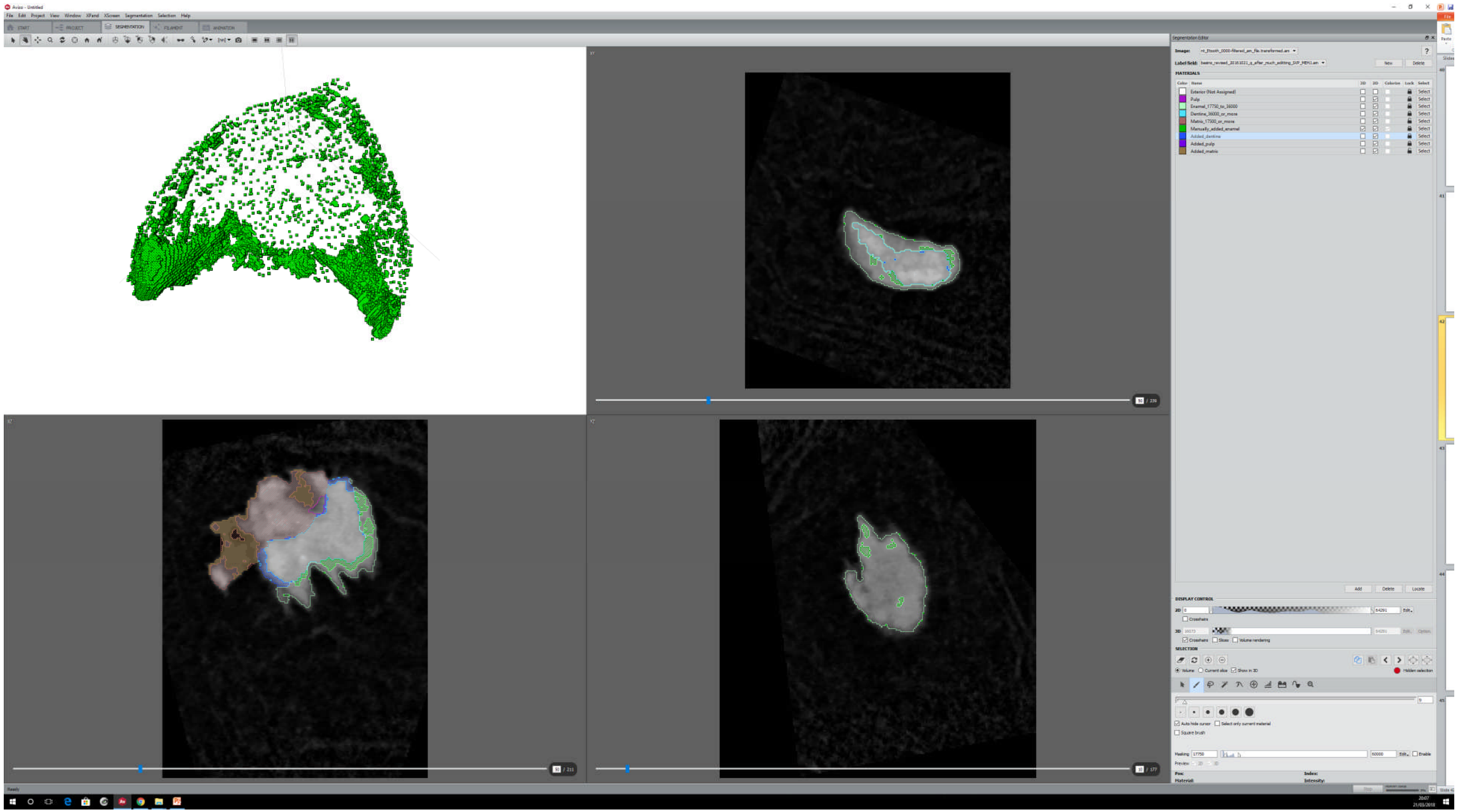

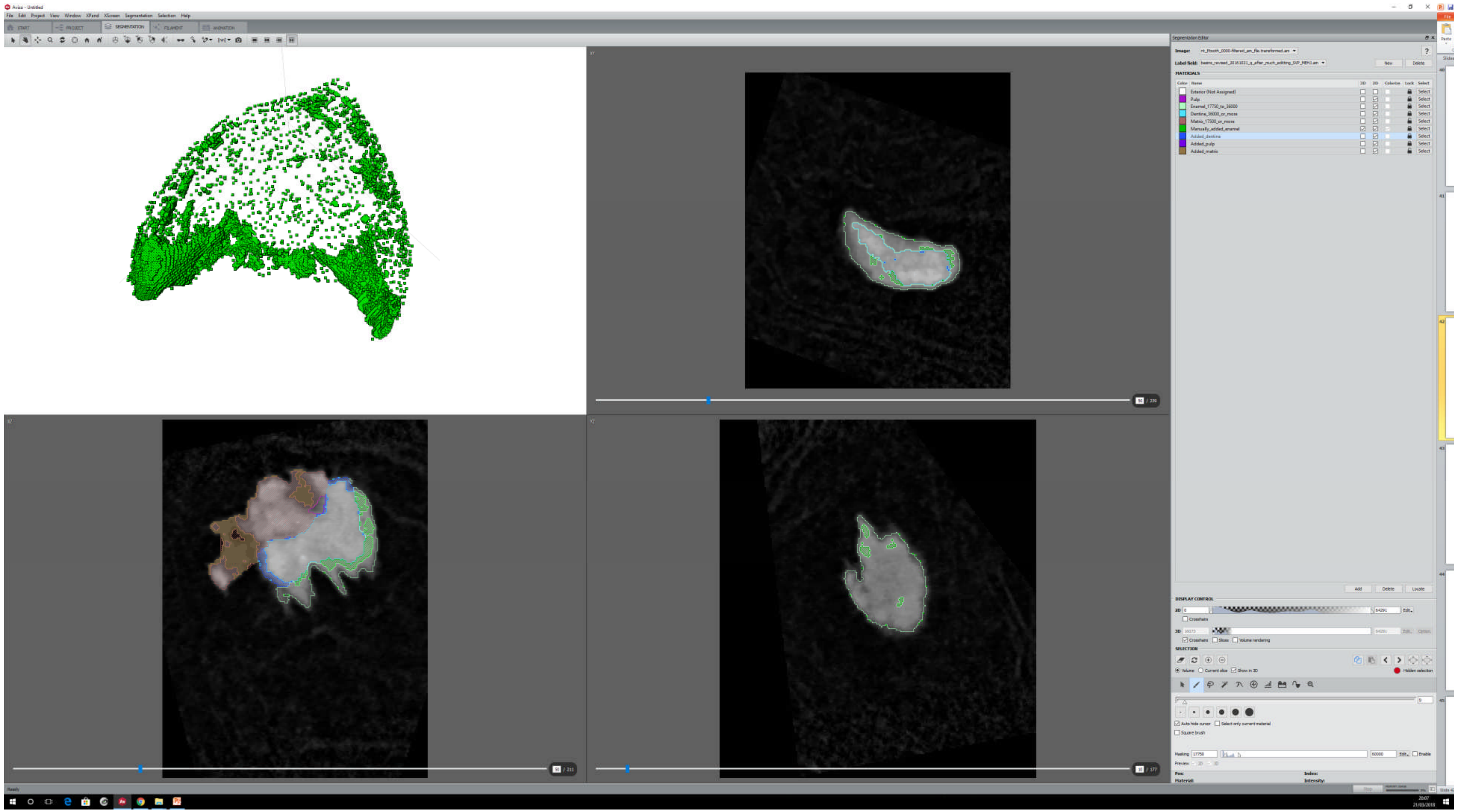
Avizo - Untitled
File Edit Project View Window XPand XScreen Segmentation Selection Help
START
PROJECT
SEGMENTATION
FILAMENT
ANIMATION

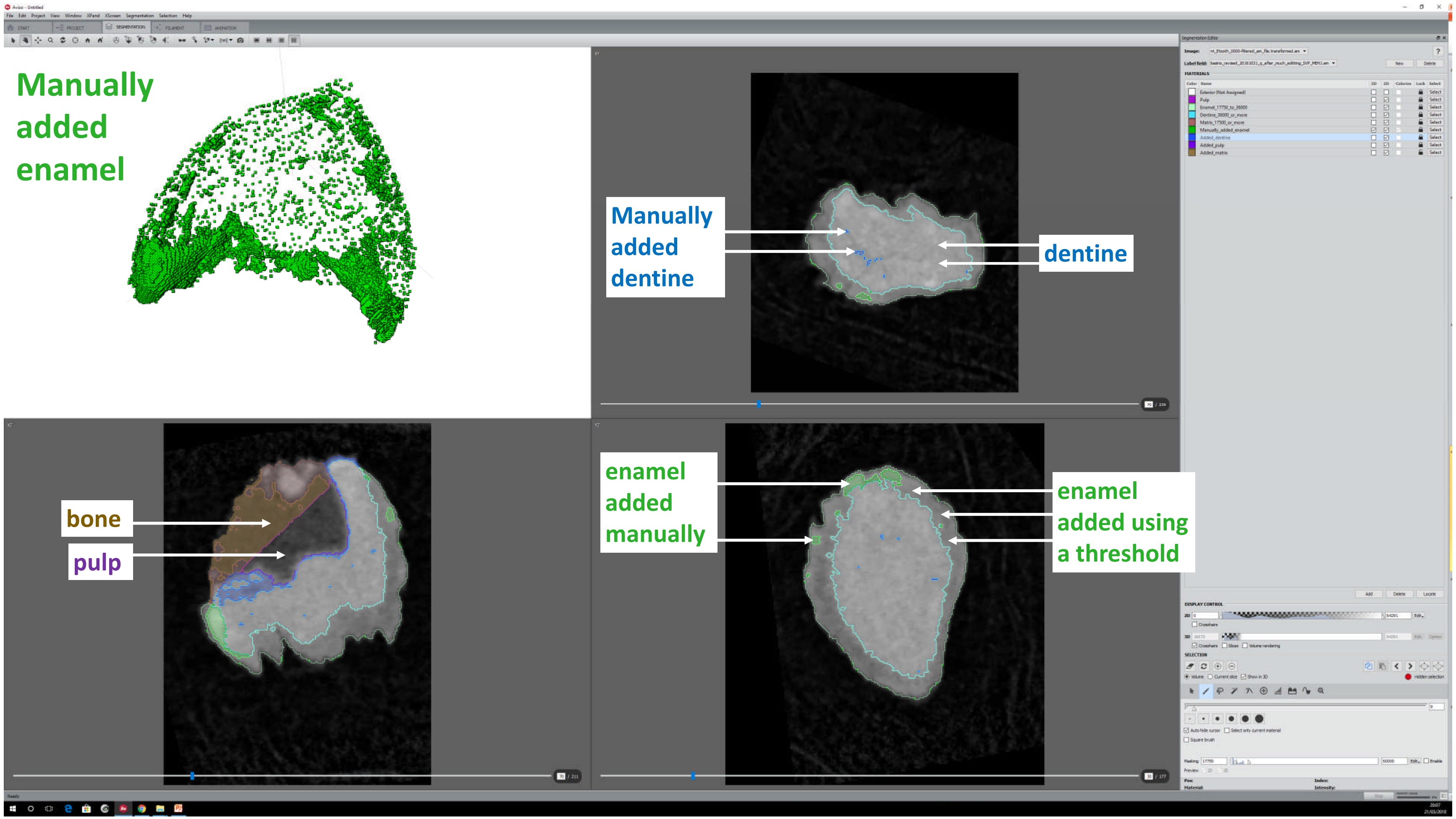

Manually added enamel
Manually added dentine
dentine
bone
pulp
enamel added manually
enamel added using a threshold

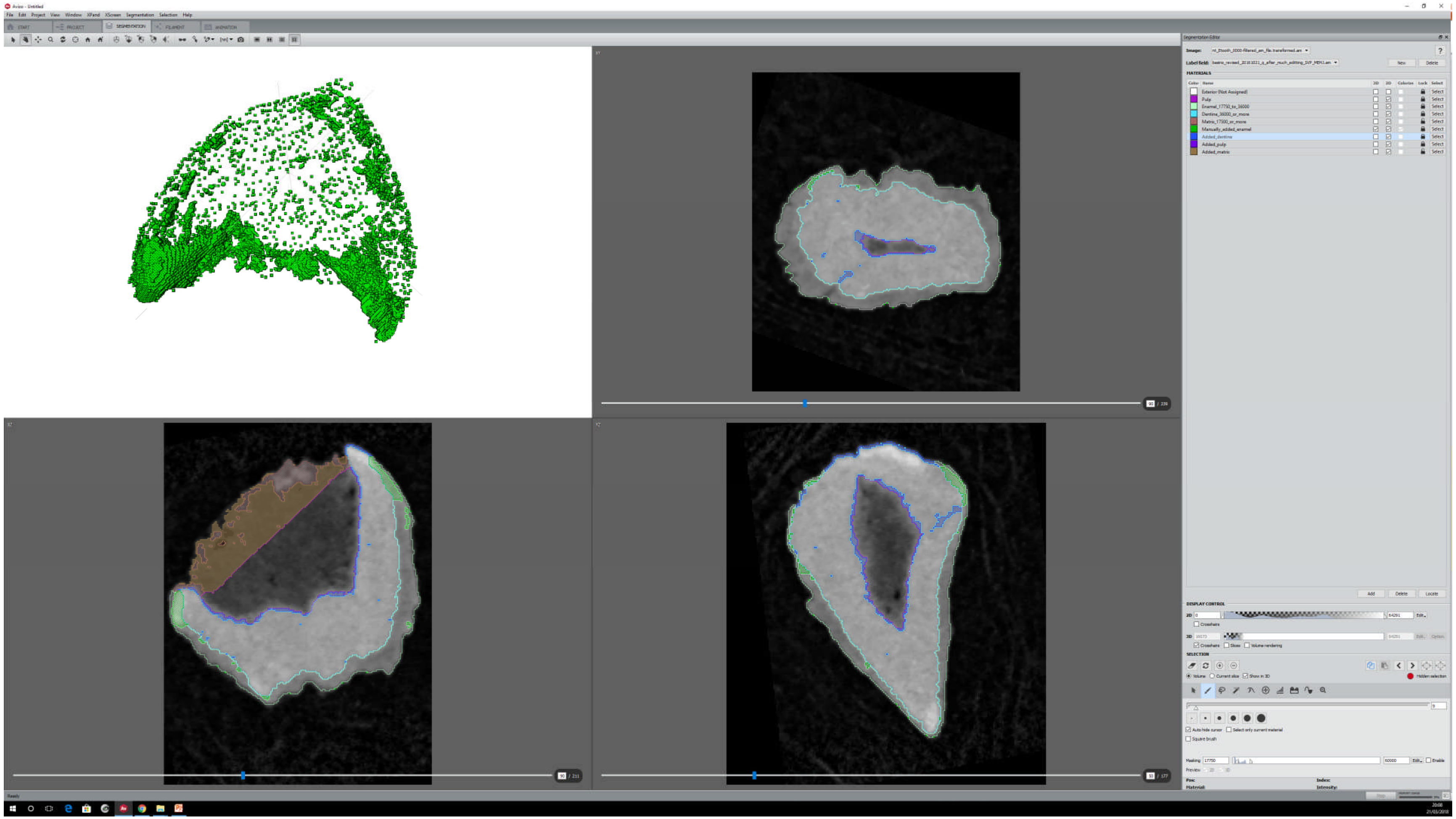

Avizo - Untitled
File Edit Project View Window XPand XScreen Segmentation Selection Help
Segmentation Editor
Image:
Label field:
New
Delete
MATERIALS
Color Name 2D 3D Colorize Lock Select
Exterior (Not Assigned)
Pulp
Enamel_17750_to_36000
Dentine_36000_or_more
Matrix_17500_or_more
Manually_added_enamel
Added_dentine
Added_pulp
Added_matrix

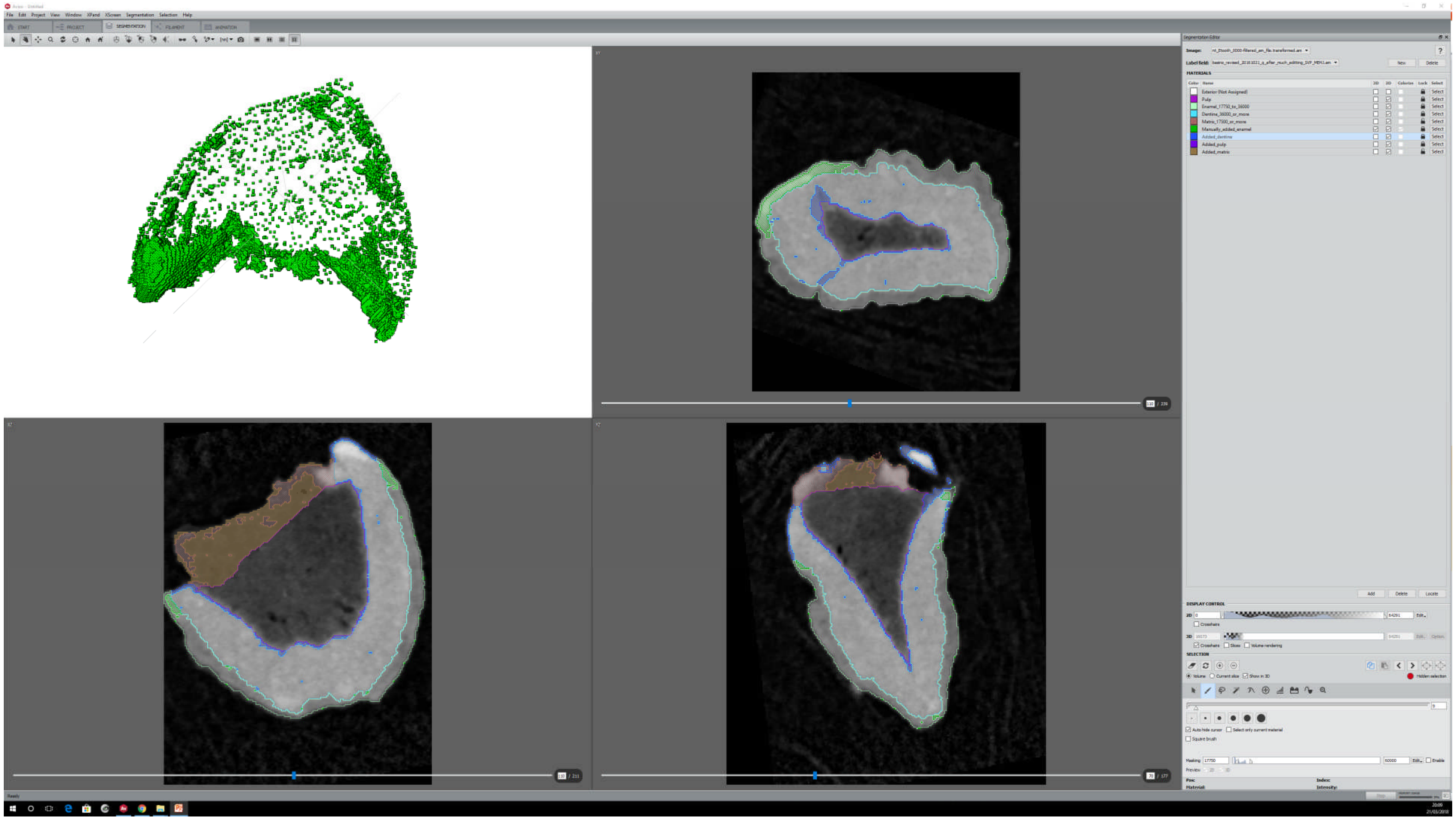

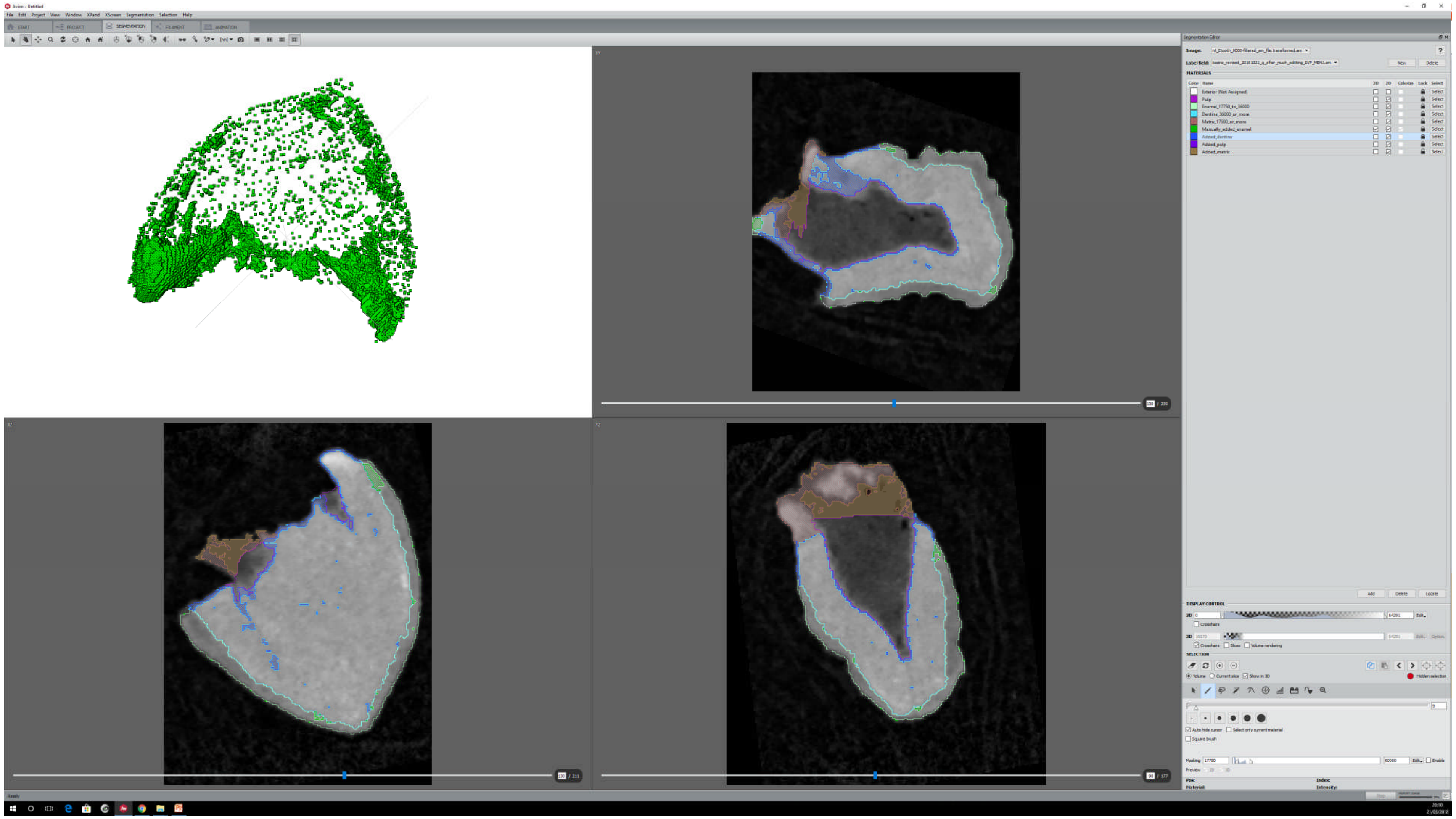
Avizo - Untitled
File Edit Project View Window XPand XScreen Segmentation Selection Help
SEGMENTATION

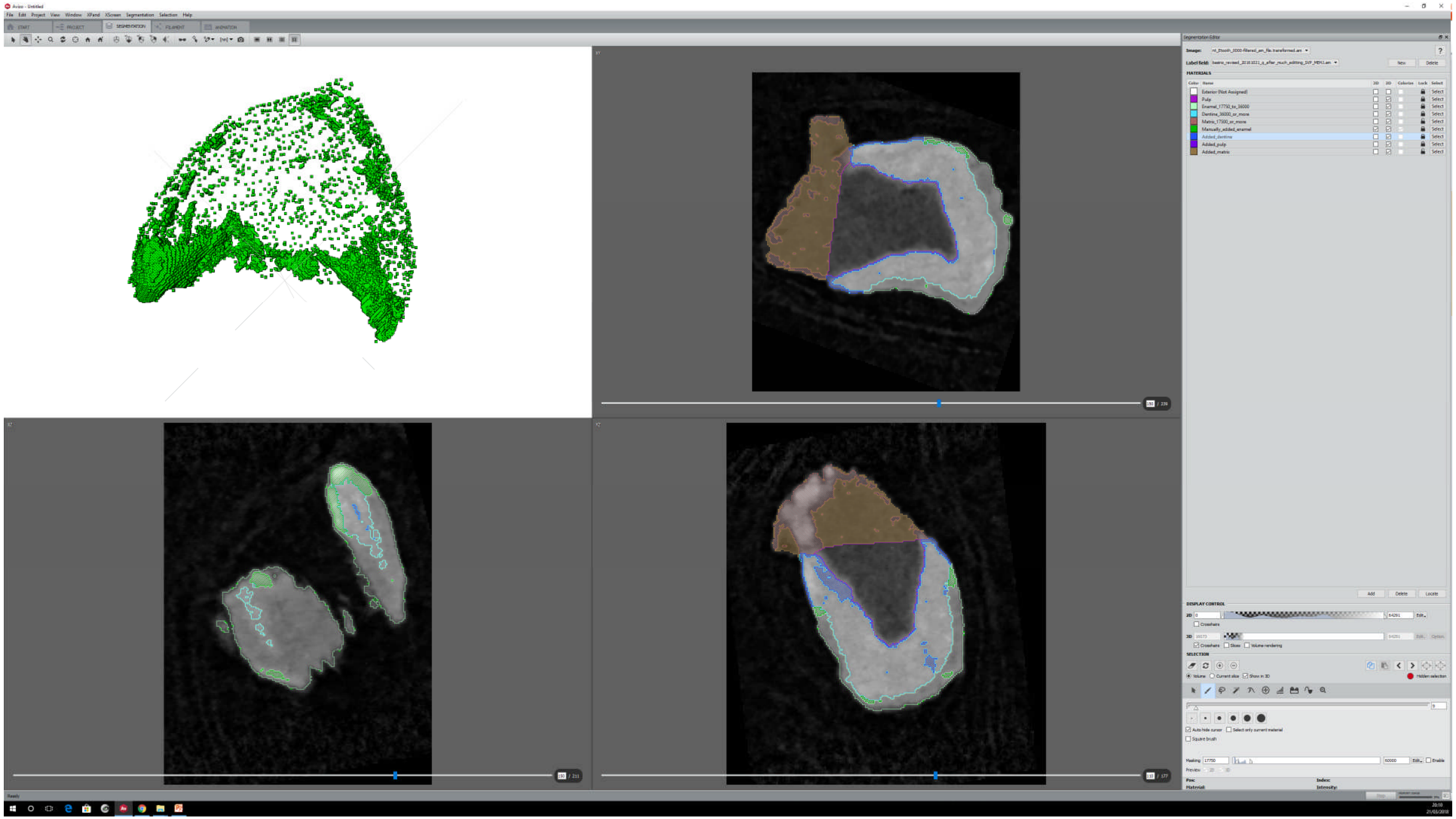

Avizo - Untitled
File Edit Project View Window XPand XScreen Segmentation Selection Help
START
PROJECT
SEGMENTATION
FILAMENT
ANIMATION
Segmentation Editor
Image:
Label field:
New
Delete
MATERIALS
Color
Name
3D
2D
Colorize
Lock
Select
Exterior (Not Assigned)
Pulp
Enamel_17750_to_36000
Dentine_36000_or_more
Matrix_17500_or_more
Manually_added_enamel
Added_dentine
Added_pulp
Added_matrix

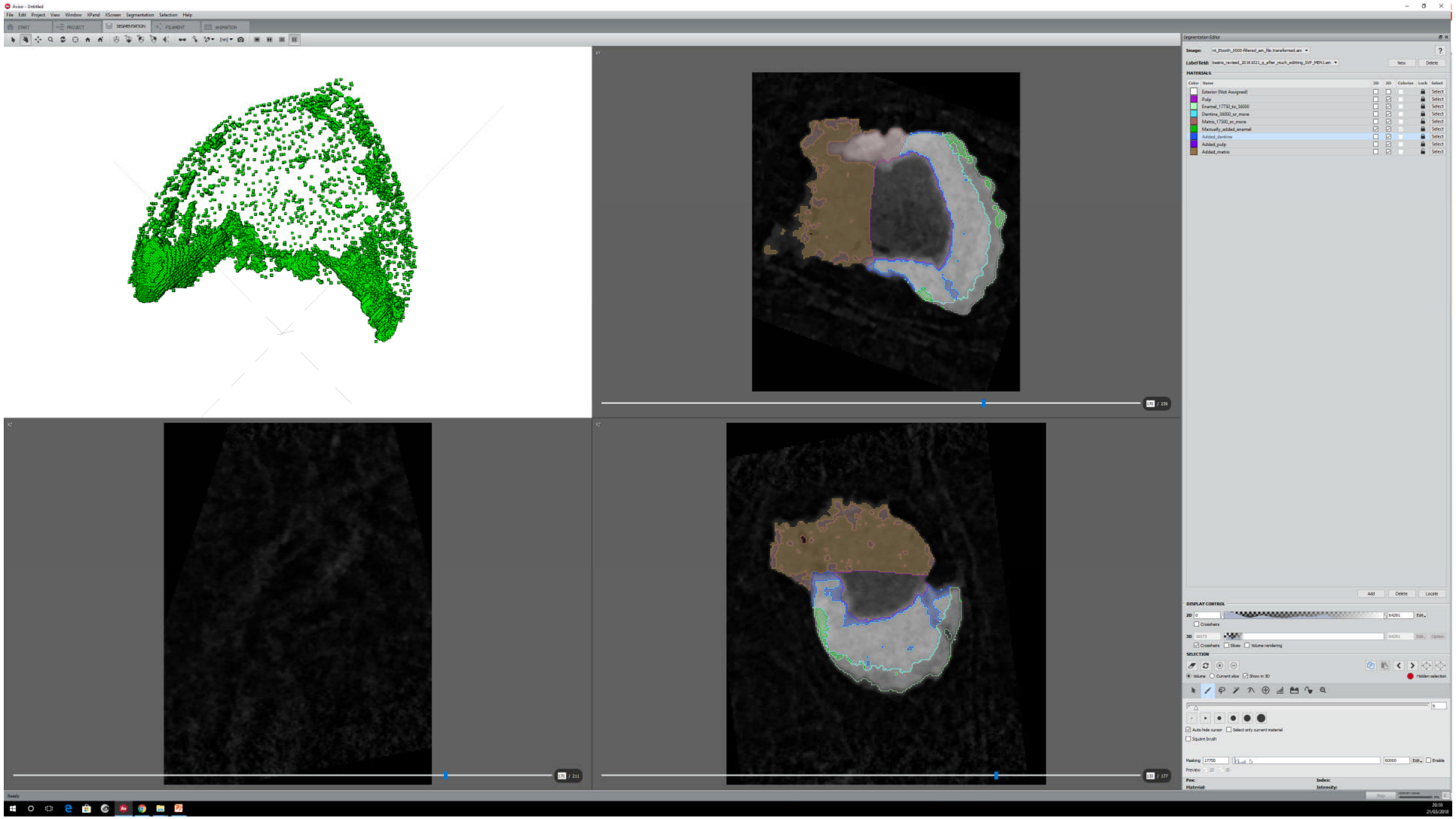

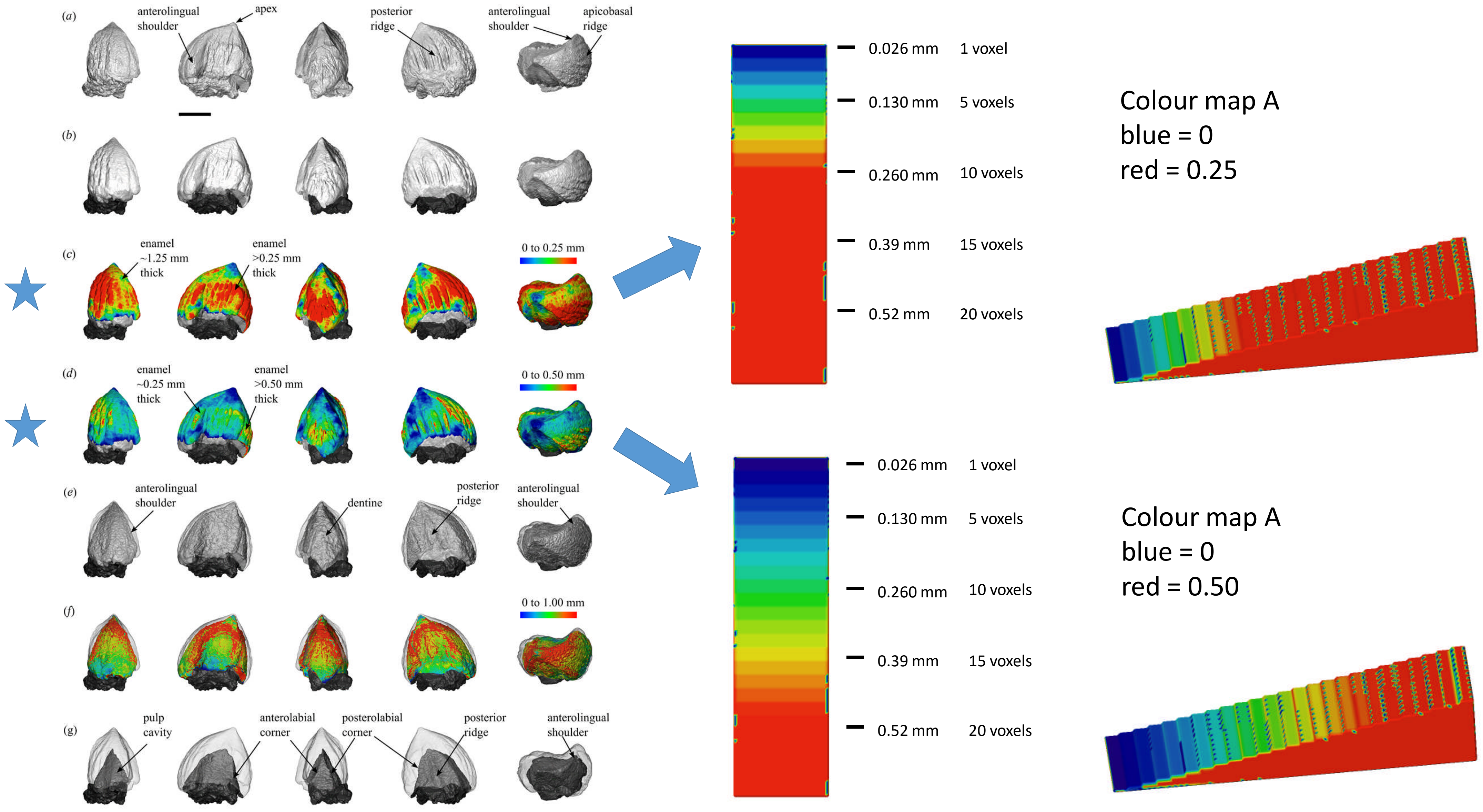
(a)
anterolingual shoulder
apex
posterior ridge
anterolingual shoulder
apicobasal ridge
(b)
(c)
enamel ~1.25 mm thick
enamel >0.25 mm thick
0 to 0.25 mm
(d)
enamel ~0.25 mm thick
enamel >0.50 mm thick
0 to 0.50 mm
(e)
anterolingual shoulder
dentine
posterior ridge
anterolingual shoulder
(f)
0 to 1.00 mm
(g)
pulp cavity
anterolabial corner
posterolabial corner
posterior ridge
anterolingual shoulder
0.026 mm 1 voxel
0.130 mm 5 voxels
0.260 mm 10 voxels
0.39 mm 15 voxels
0.52 mm 20 voxels
Colour map A
blue = 0
red = 0.25
0.026 mm 1 voxel
0.130 mm 5 voxels
0.260 mm 10 voxels
0.39 mm 15 voxels
0.52 mm 20 voxels
Colour map A
blue = 0
red = 0.50

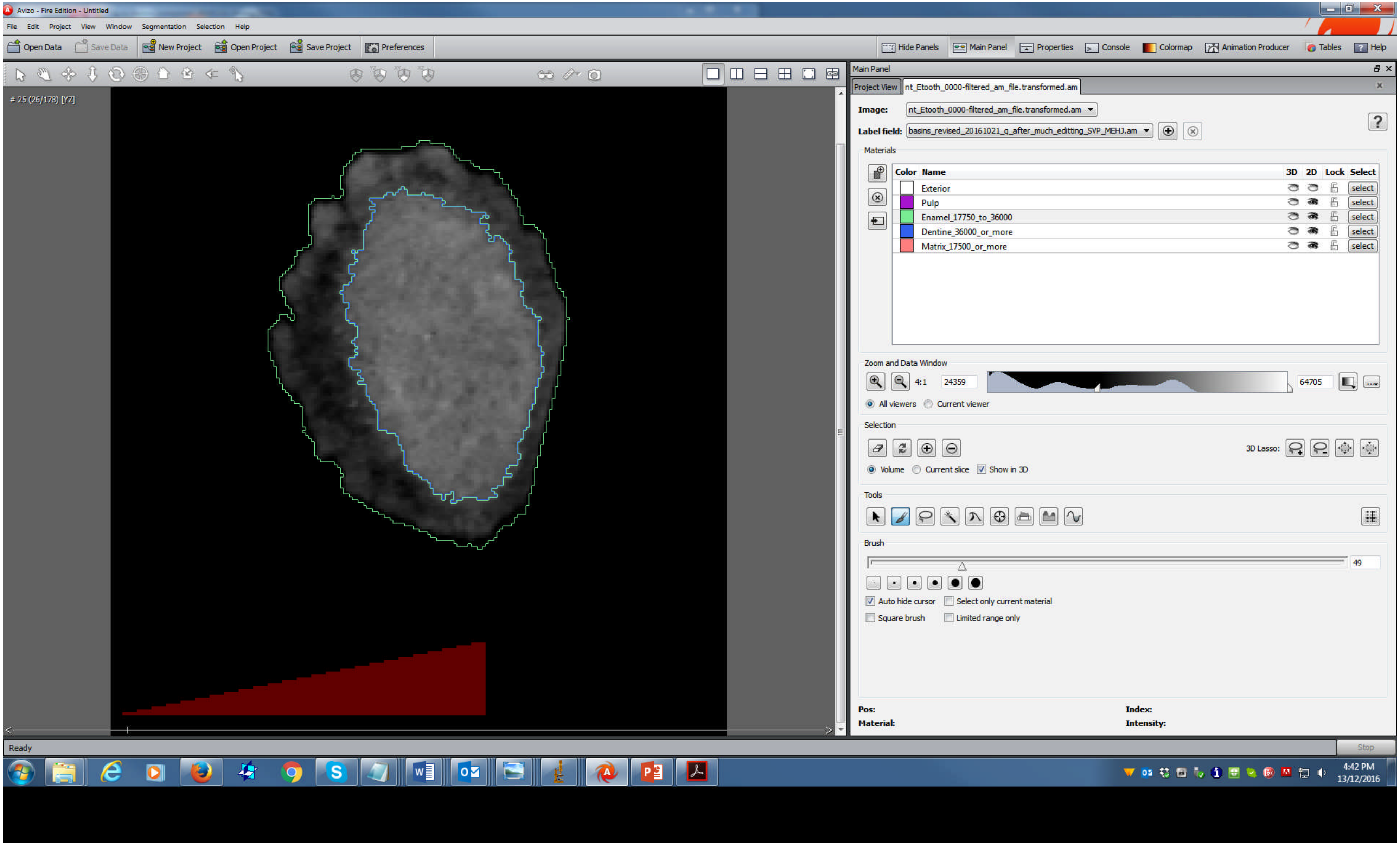
Avizo - Fire Edition - Untitled
File Edit Project View Window Segmentation Selection Help
Open Data Save Data New Project Open Project Save Project Preferences
Hide Panels Main Panel Properties Console Colormap Animation Producer Tables Help
25 (26/178) [YZ]
Main Panel
Project View
nt_Etooth_0000-filtered_am_file.transformed.am
Image: nt_Etooth_0000-filtered_am_file.transformed.am
Label field: basins_revised_20161021_q_after_much_editting_SVP_MEHJ.am
Materials
Color Name 3D 2D Lock Select
Exterior select
Pulp select
Enamel_17750_to_36000 select
Dentine_36000_or_more select
Matrix_17500_or_more select
Zoom and Data Window
4:1 24359 64705
All viewers Current viewer
Selection
3D Lasso:
Volume Current slice Show in 3D
Tools
Brush
49
Auto hide cursor Select only current material
Square brush Limited range only
Pos: Index:
Material: Intensity:
Ready
Stop
4:42 PM
13/12/2016

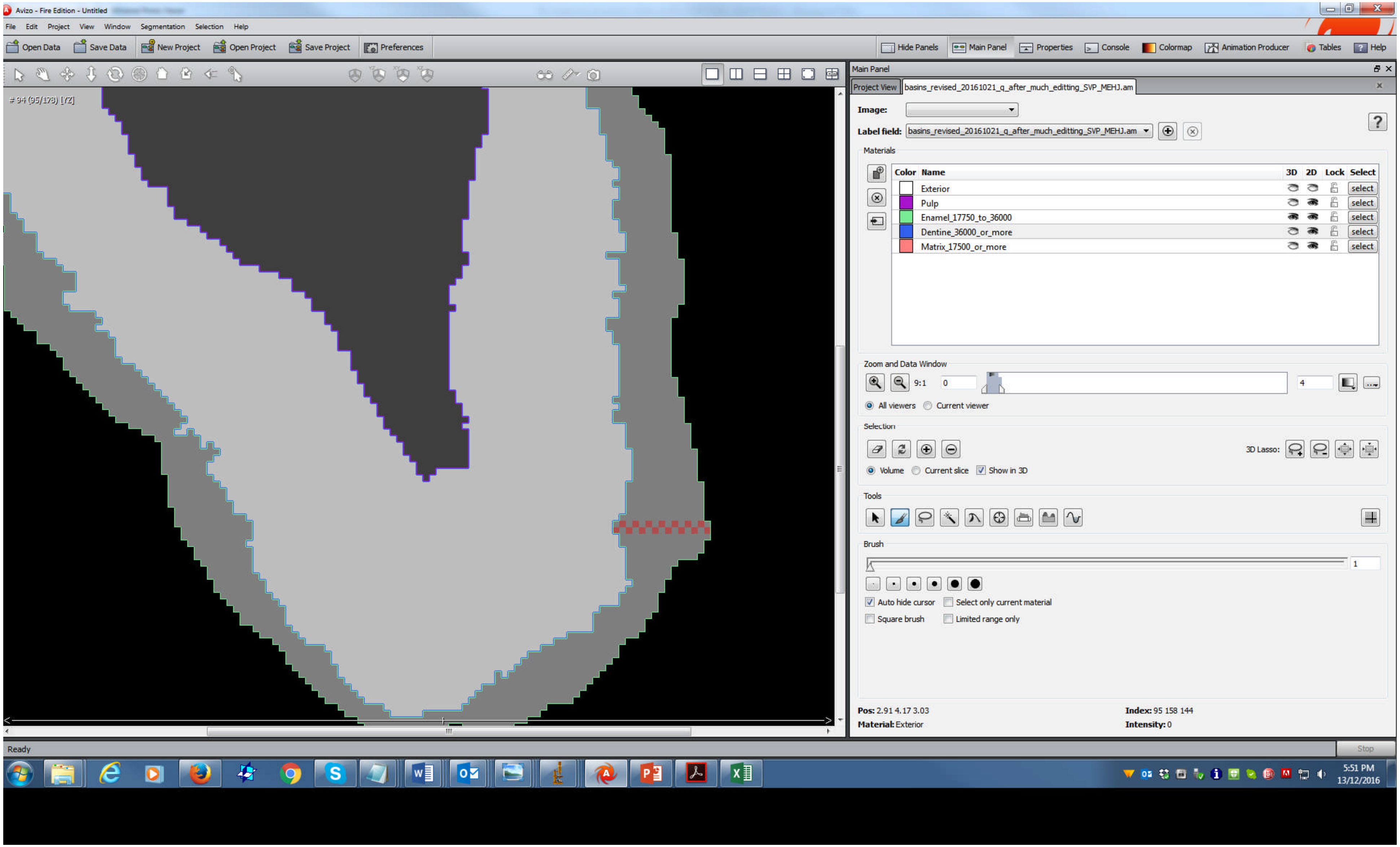
Avizo - Fire Edition - Untitled
File Edit Project View Window Segmentation Selection Help
Open Data
Save Data
New Project
Open Project
Save Project
Preferences
Hide Panels
Main Panel
Properties
Console
Colormap
Animation Producer
Tables
Help
Main Panel
Project View
basins_revised_20161021_q_after_much_editting_SVP_MEHJ.am
Image:
Label field: basins_revised_20161021_q_after_much_editting_SVP_MEHJ.am
Materials
Color Name 3D 2D Lock Select
Exterior select
Pulp select
Enamel_17750_to_36000 select
Dentine_36000_or_more select
Matrix_17500_or_more select
Zoom and Data Window
9:1
0
4
All viewers
Current viewer
Selection
3D Lasso:
Volume
Current slice
Show in 3D
Tools
Brush
1
Auto hide cursor
Select only current material
Square brush
Limited range only
Pos: 2.91 4.17 3.03
Index: 95 158 144
Material: Exterior
Intensity: 0
Ready
Stop
5:51 PM
13/12/2016
