# Supplementary material for: Neutron scanning reveals unexpected complexity in the enamel thickness of an herbivorous Jurassic reptile
Source: J R Soc Interface. 2018 Jun 13;15(143):20180039. doi: 10.1098/rsif.2018.0039 (PMC6030635; doi:10.1098/rsif.2018.0039)
Supplement: SI Additional Figures [file rsif20180039supp2.docx]

## Supplementary Information – additional figures


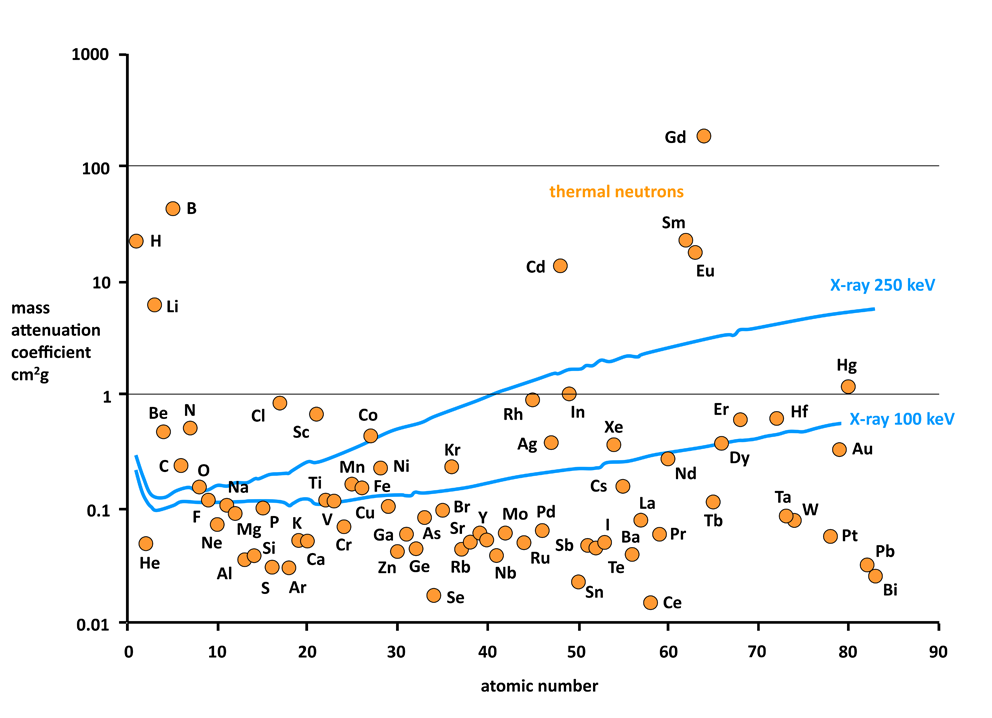


### SI Figure 1. The attenuation coefficients of X-rays and neutrons. Redrawn from Schwarz 2005 [59]: figure 1.


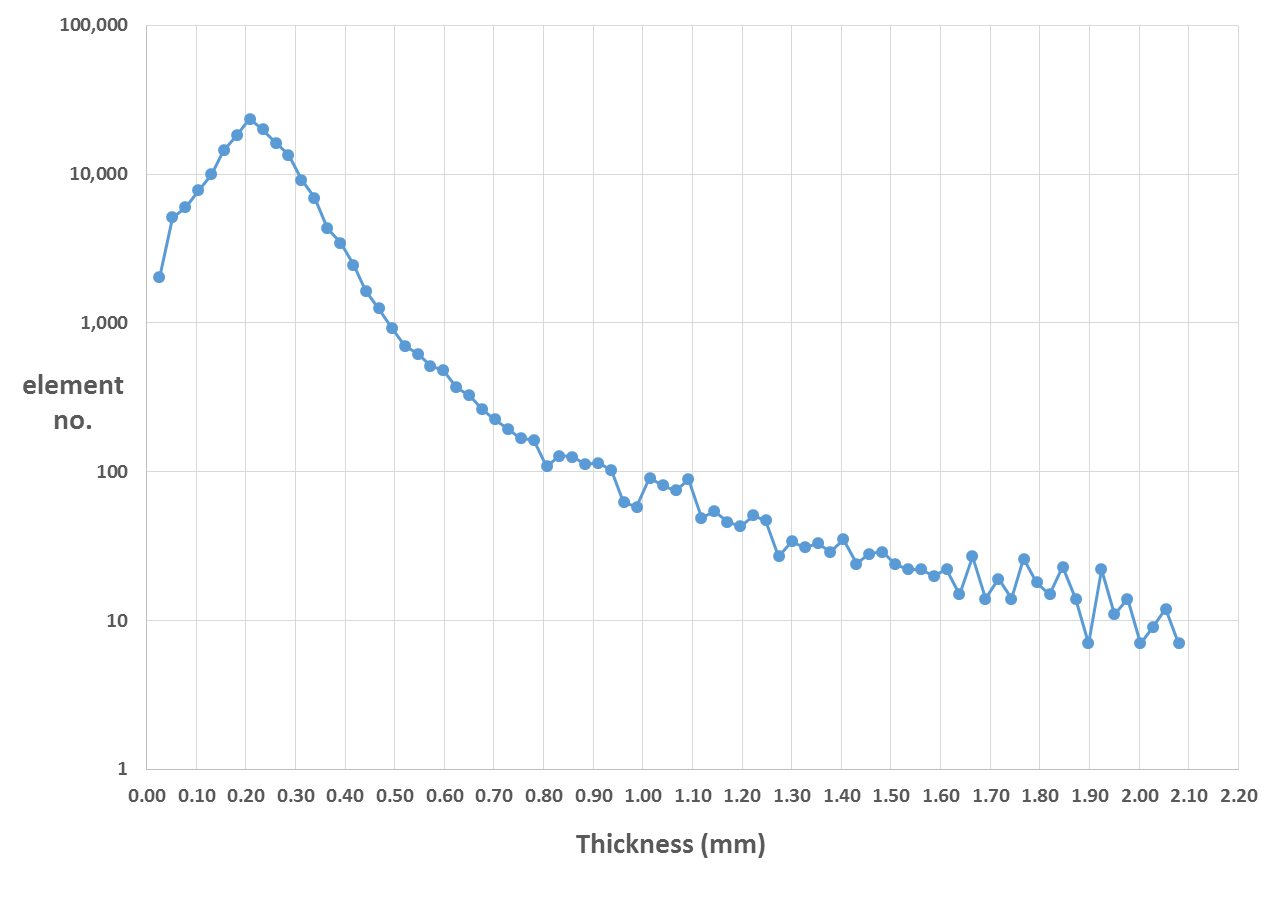


### SI Figure 2. Enamel thickness frequency according to the unsmoothed surface element segmentation of the neutron CT dataset. Similar to figure 5 but showing the full dataset up to a thickness of 2.2 mm and with the y axis in log scale.


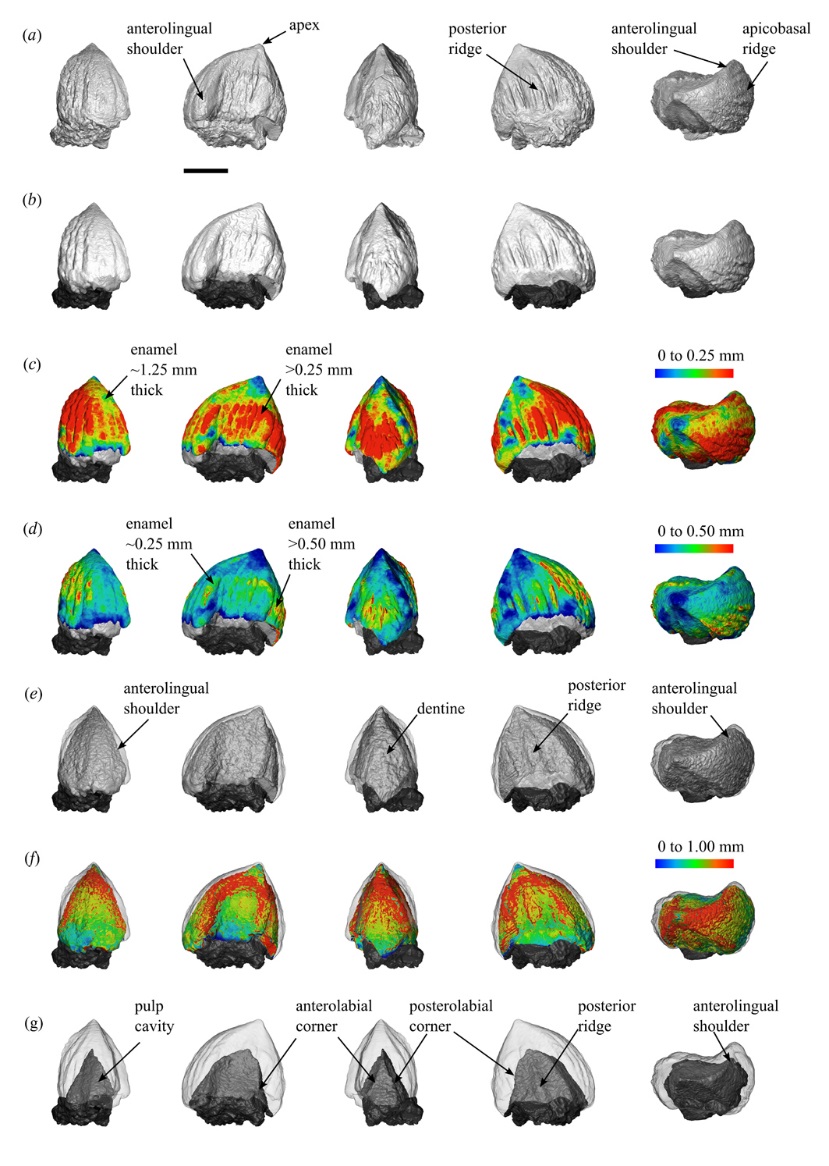


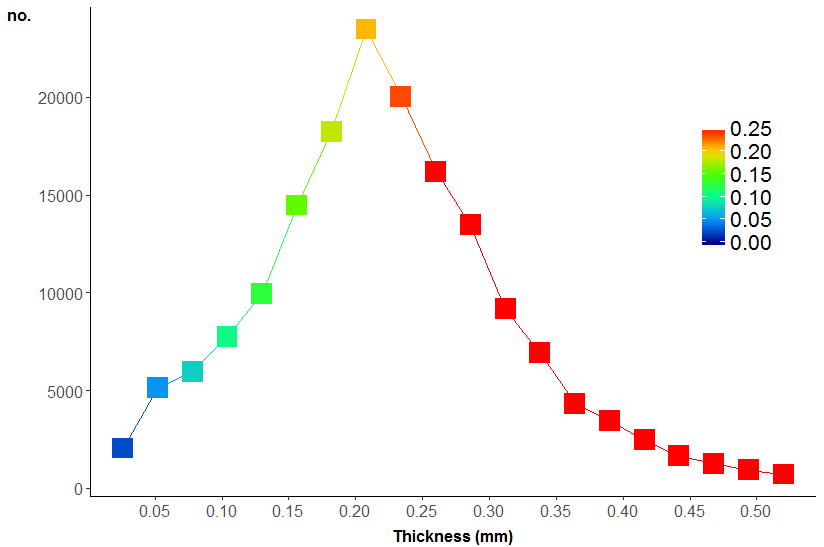


###
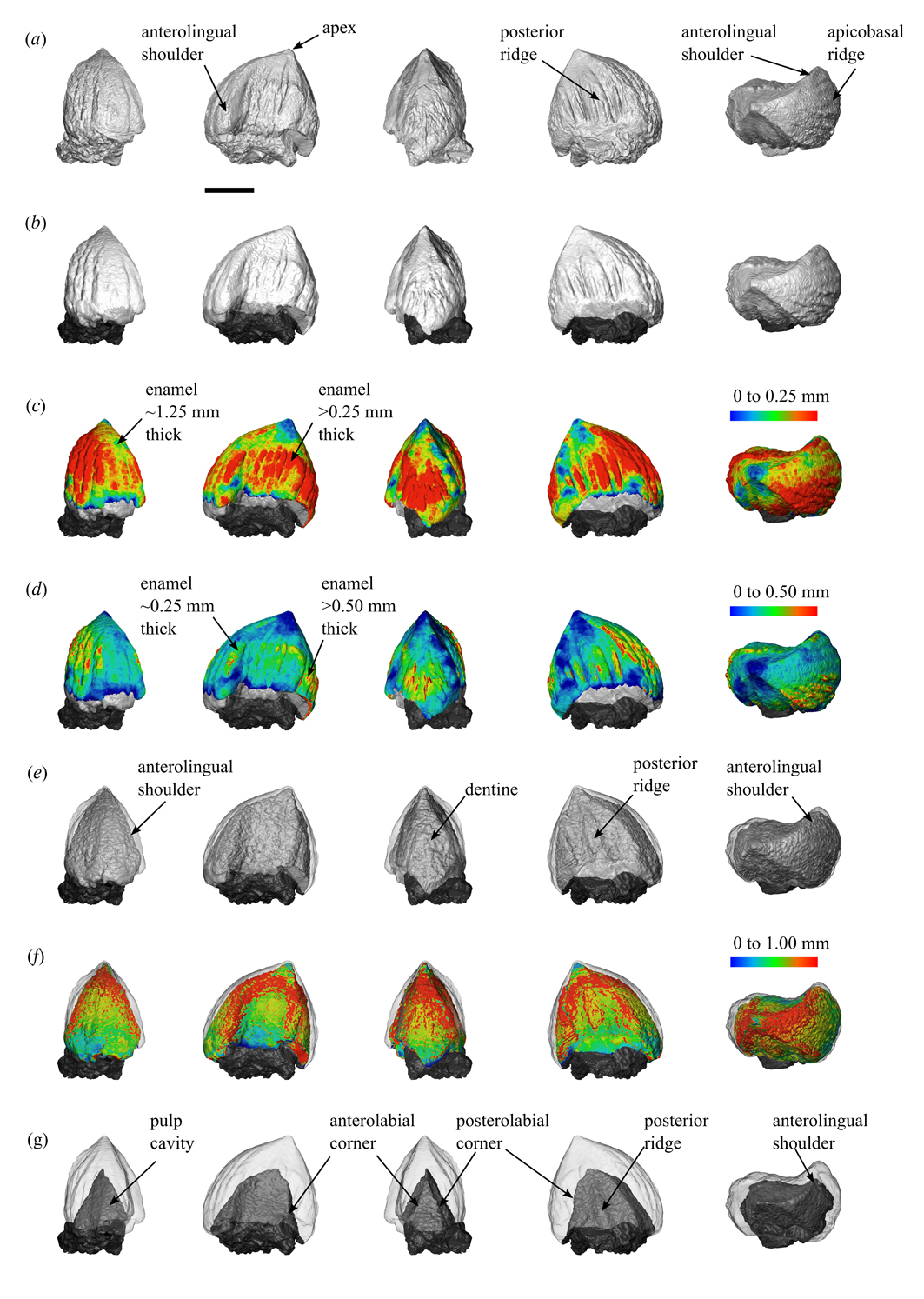
SI Figure 3. Enamel thickness frequency according to the unsmoothed surface element segmentation of the neutron CT dataset. Similar to figure 5 but with a different colour scale and complimentary images from figure 6.


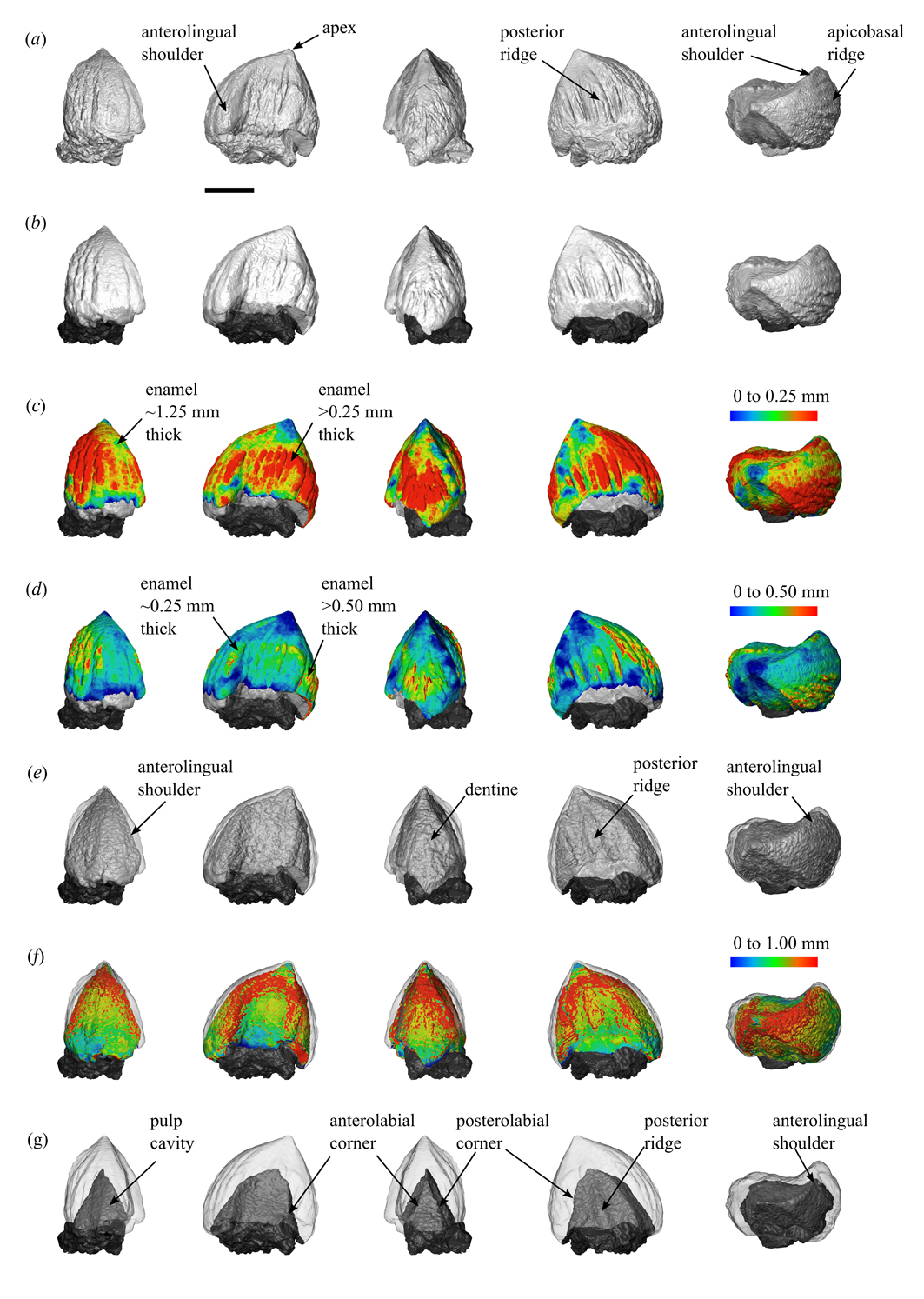


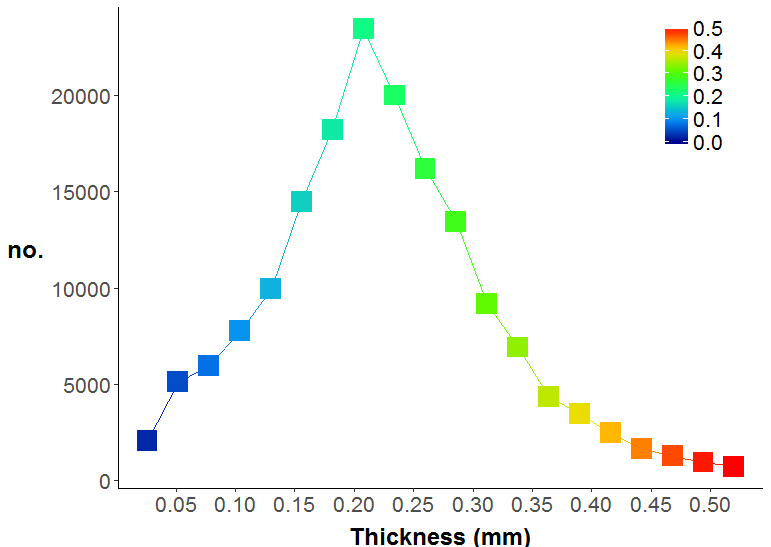


###
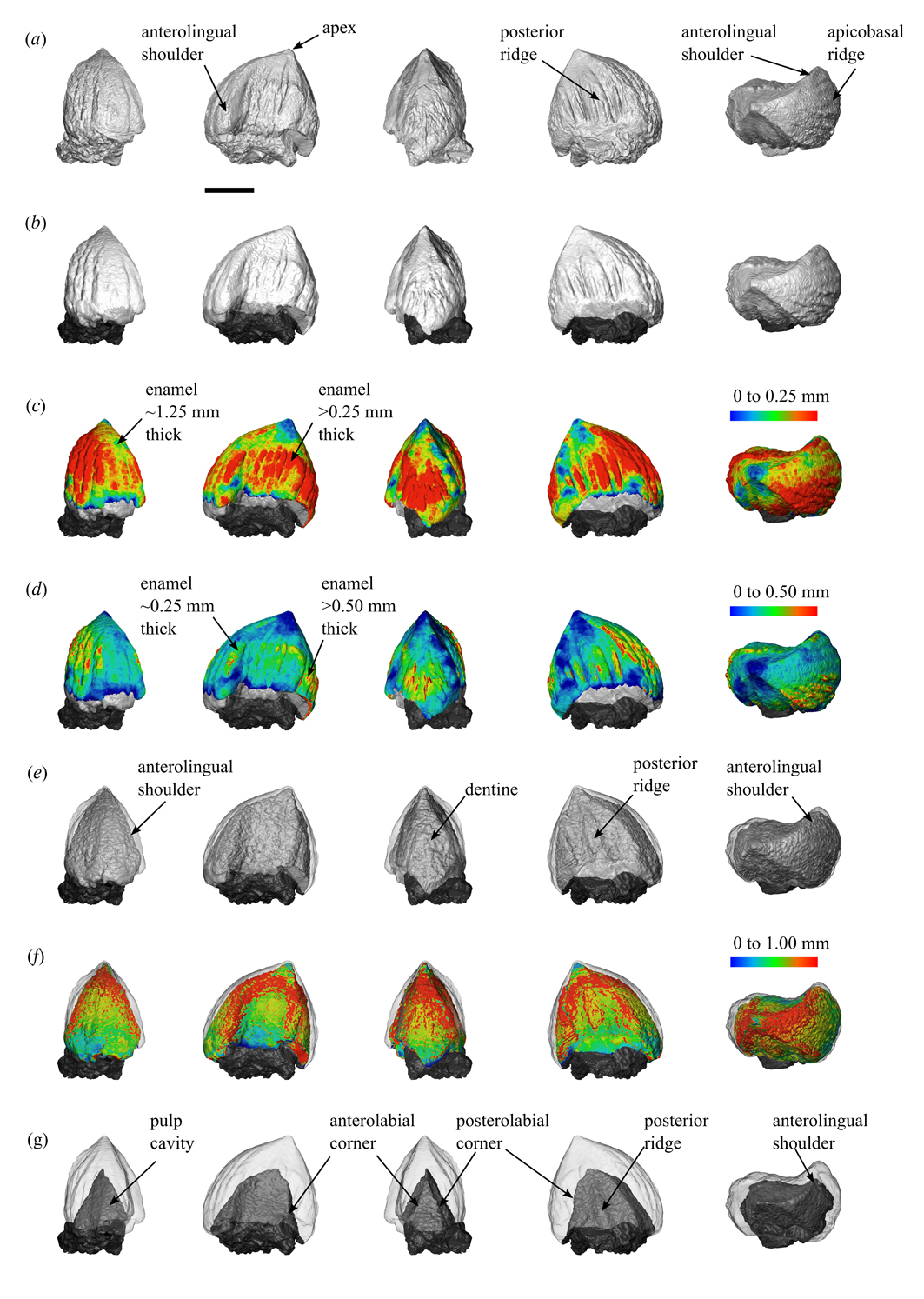
SI Figure 4. Enamel thickness frequency according to the unsmoothed surface element segmentation of the neutron CT dataset. The same as figure 5 but with complimentary images from figure 6.


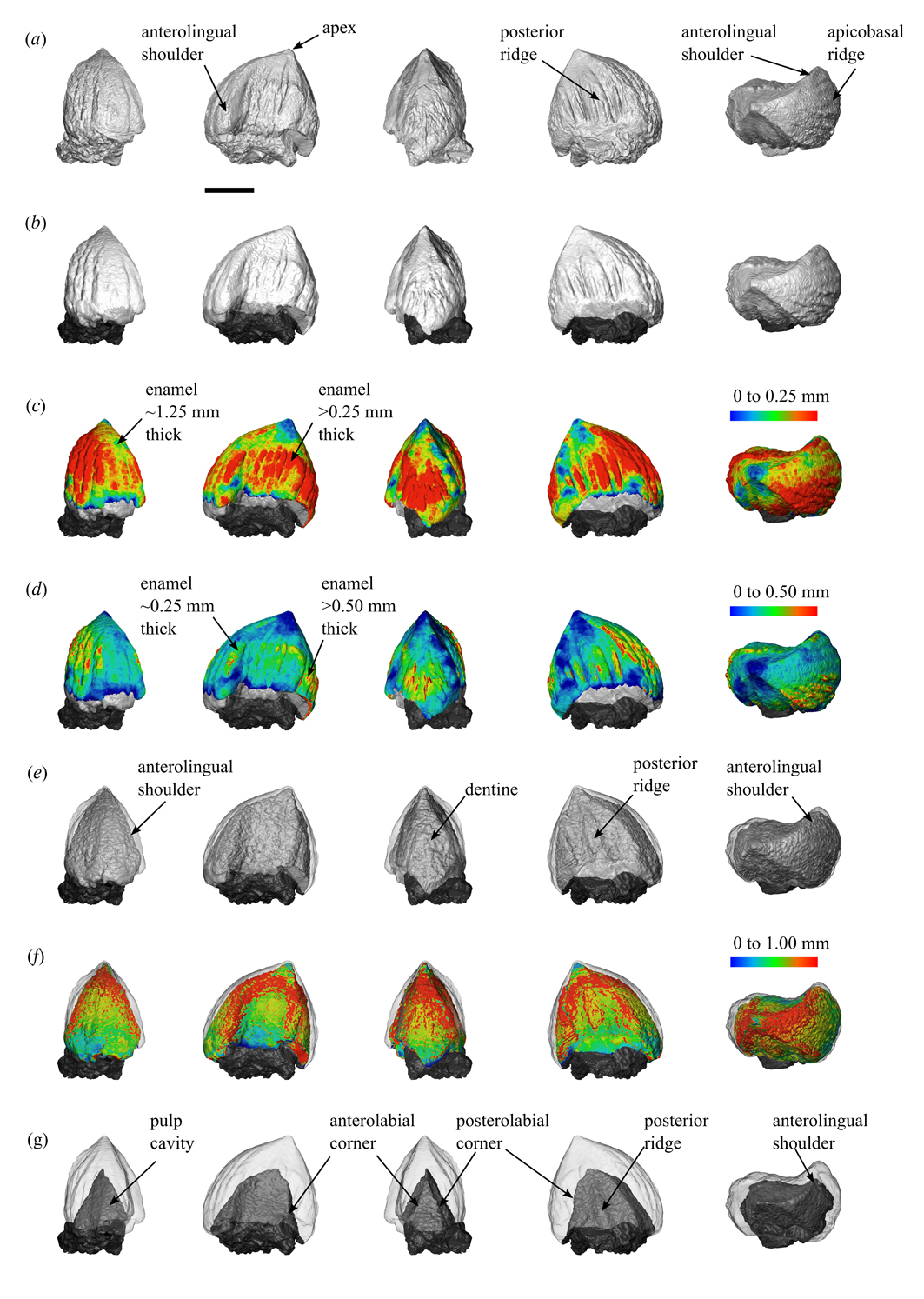


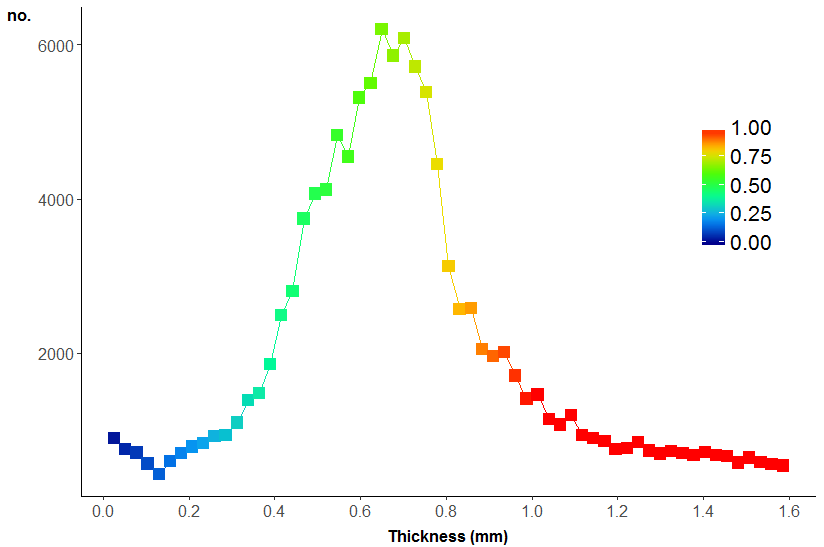

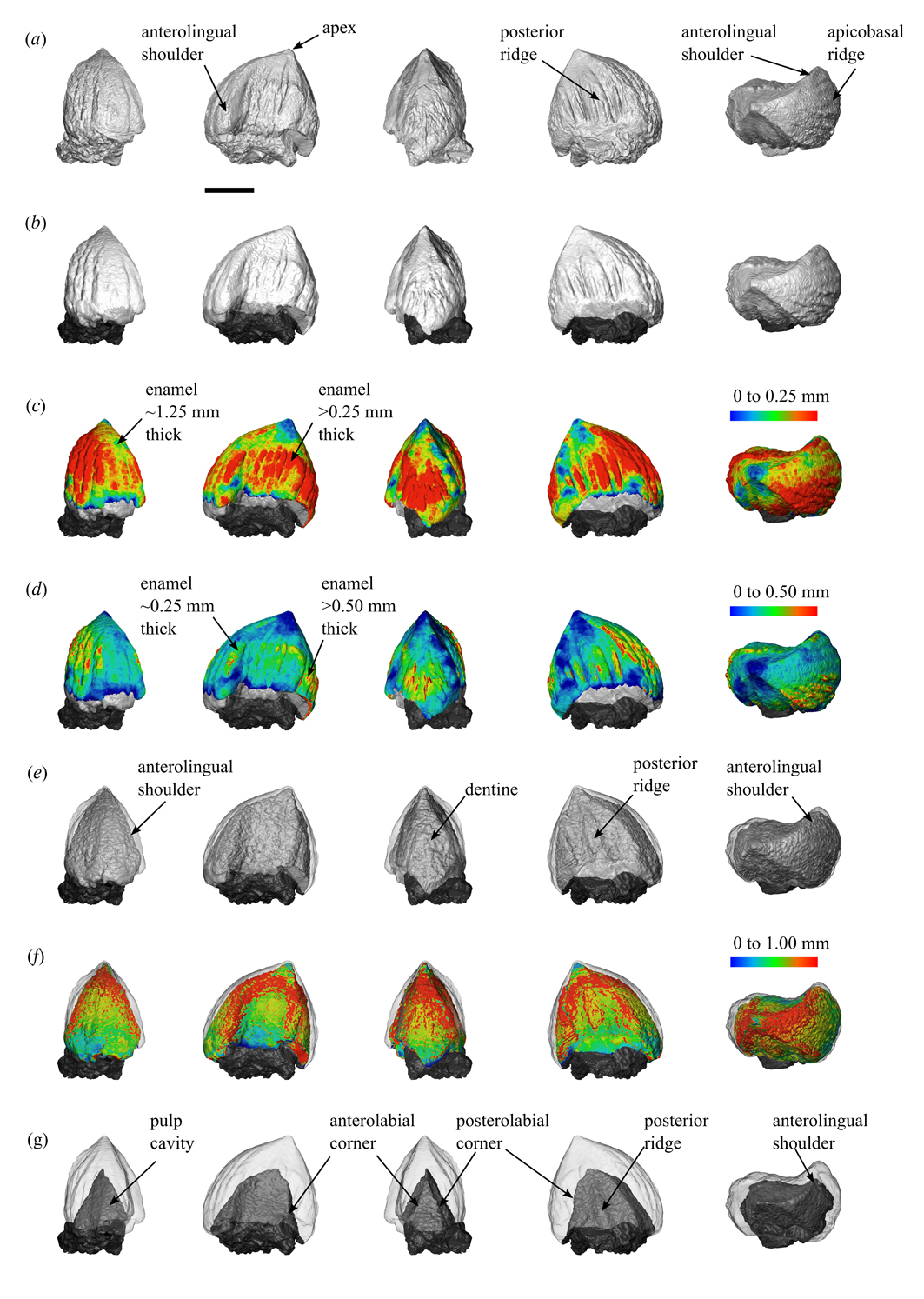


### SI Figure 5. Dentine thickness frequency according to the unsmoothed surface element segmentation of the neutron CT dataset.


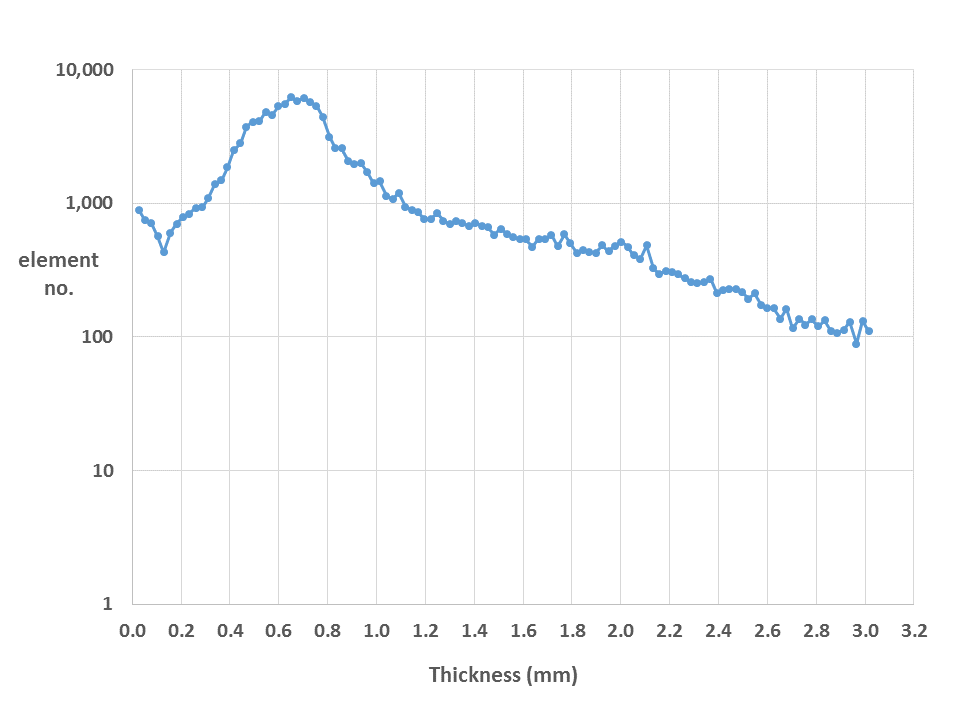


### SI Figure 6. Dentine thickness frequency according to the unsmoothed surface element segmentation of the neutron CT dataset. Showing the full dataset up to a thickness of 3.2 mm with the y axis in log scale.


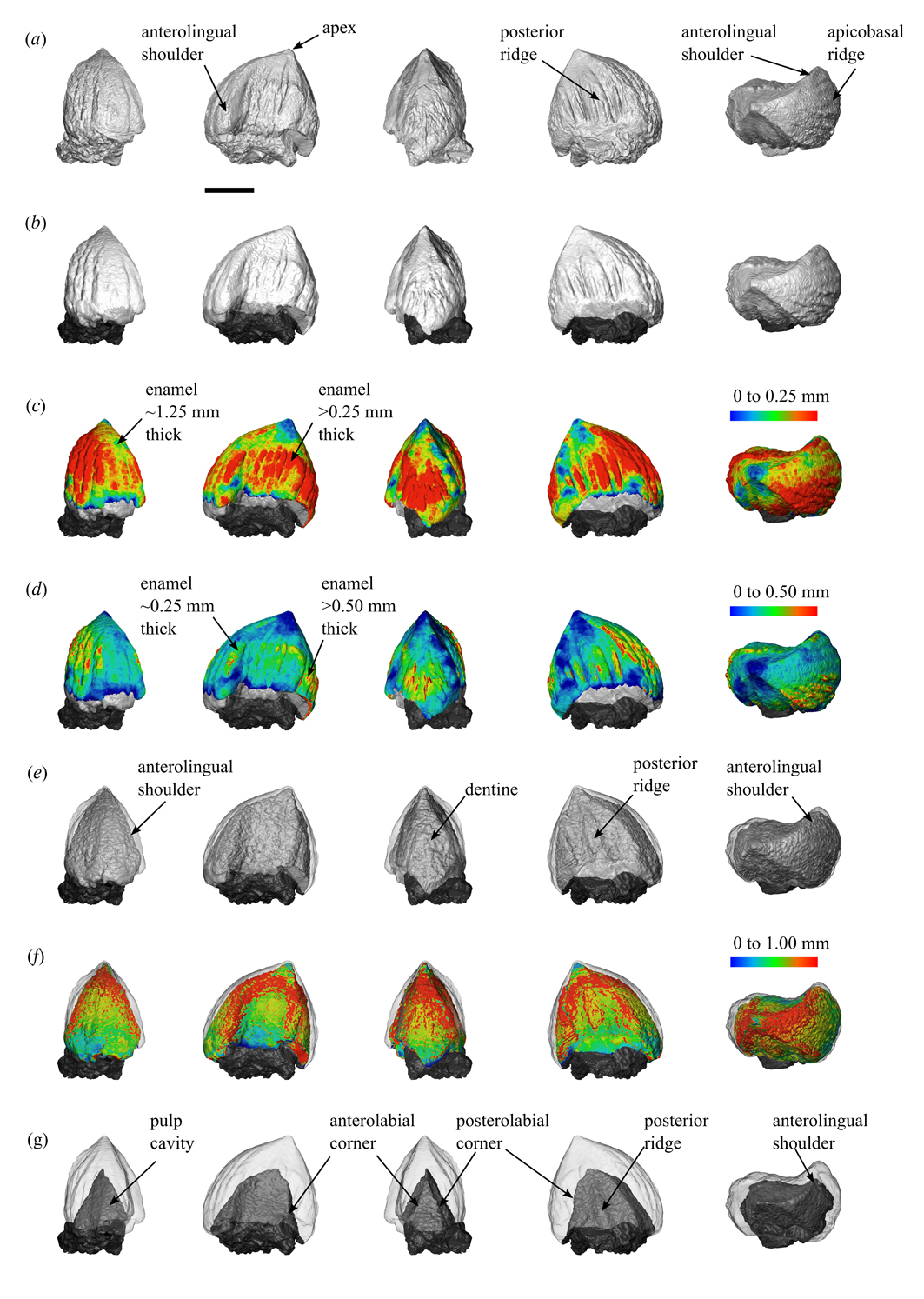

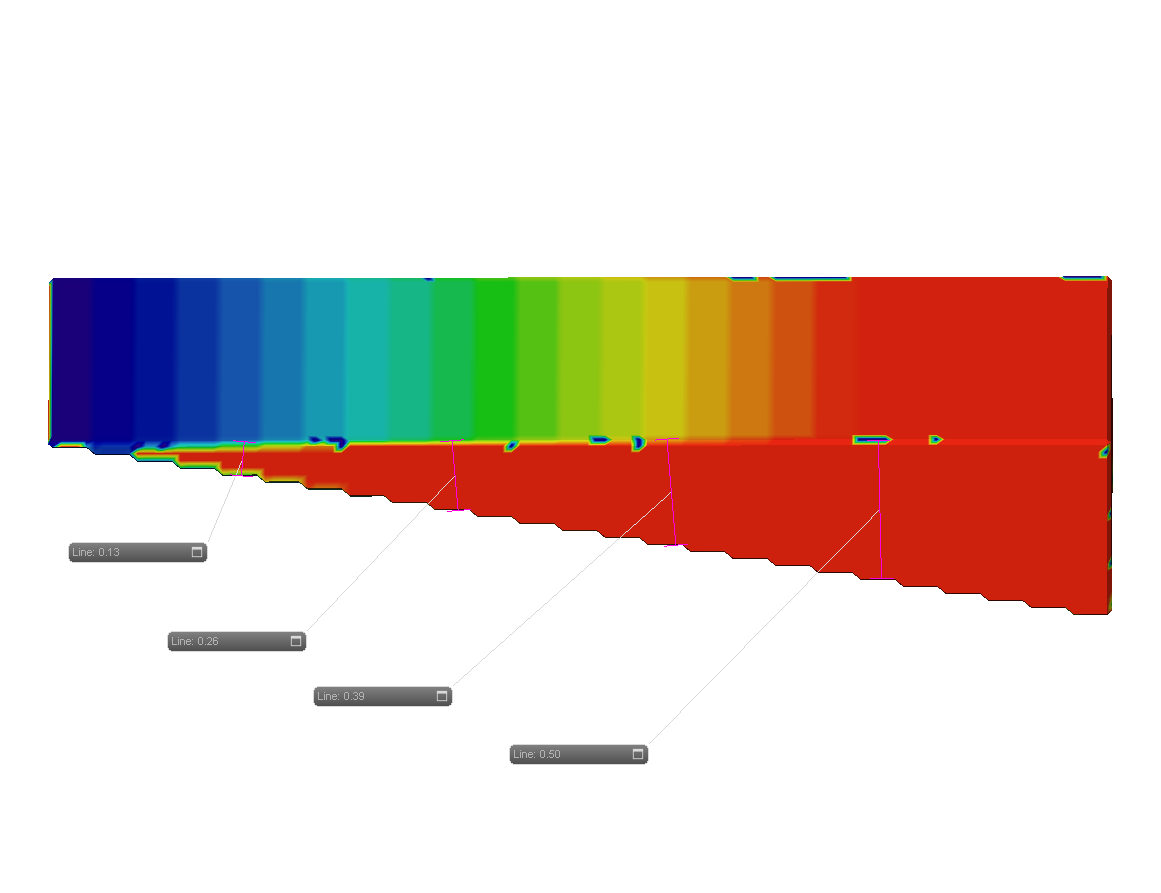


### SI Figure 7. Outer Enamel Surface of the tooth colour coded for enamel thickness next to a stepped model built in Avizo to test the reliability of the colour coding by thickness. Each step is 0.026 mm. Thus, five steps are 0.13 mm thick.
