# Supplementary material for: Neutron scanning reveals unexpected complexity in the enamel thickness of an herbivorous Jurassic reptile
Source: J R Soc Interface. 2018 Jun 13;15(143):20180039. doi: 10.1098/rsif.2018.0039 (PMC6030635; doi:10.1098/rsif.2018.0039)
Supplement: SI Table 1 [file rsif20180039supp3.docx]

### SI Table 1. Tooth dimension for the teeth of an adult *Sphenodon* (specimen SAMA 70524). Measured in Avizo 8.1 from a model based on micro CT data. Micro-CT scanning was performed with a Phoenix Nanotom m (GE Sensing & Inspection Technologies GmbH, Wunstorf, Germany) operated using xs control and Phoenix datos|x acquisition software (both GE Sensing & Inspection Technologies). The preserved Tuatara specimen was wrapped in bubble wrap to fit into a PVC pipe of 16 cm diameter to ensure no sample movement during micro-CT scanning. The sample was scanned at two resolutions, two scans at 25 μm for the head and 3 scans at 62 μm for the entire specimen. Scans were run for 10 minutes in fast scan mode (timing = 500 ms, av = 1, skip = 0) at 100 kV and 400 μA, collecting 1199 X-ray projections of the sample through 360 degrees of rotation. A molybdenum target was used with a 0.5 mm Al filter to maximize contrast in the specimen. Volume reconstruction of the micro-CT data was performed using Phoenix datos|x reconstruction software (GE Sensing & Inspection Technologies) and data was exported as 32-bit float volume files. This allowed the separate scans to be stitched together using Avizo (FEI) so that a separate volume files for the head and entire specimen were produced. The authors acknowledge the support of the Trace Analysis for Chemical, Earth and Environmental Sciences (TrACEES) platform from the Melbourne Collaborative Infrastructure Research Program at the University of Melbourne and thank Dr Jay Black (School of Earth Sciences) for operating the micro-CT scanner and processing data.

| **Tooth** | **side** | **tooth no. from back** | **mesiodistal length (mm)** | **mesiodistal tooth dimension (mm)** | **crown radius (mm)** | **apicobasal height**  **(mm)** | **h/R** | **wear?** |
| --- | --- | --- | --- | --- | --- | --- | --- | --- |
| SAMA 70524.L1 | Left | 1 | na | na | na | na | na | no, but developing |
| **SAMA 70524.L2** | **Left** | **2** | **1.87** | **1.64** | **0.88** | **1.714** | **1.95** | **no** |
| SAMA 70524.L3 | Left | 3 | 2.21 | 1.65 | 0.97 | 2.136 | 2.21 | yes |
| SAMA 70524.L4 | Left | 4 | 2.43 | 1.60 | 1.01 | 1.931 | 1.92 | yes |
| SAMA 70524.L5 | Left | 5 | 2.07 | 1.36 | 0.86 | 1.602 | 1.87 | yes |
| SAMA 70524.L6 | Left | 6 | 1.61 | 1.36 | 0.74 | 1.623 | 2.19 | yes |
| **SAMA 70524.R1** | **Right** | **1** | **1.74** | **1.82** | **0.89** | **2.069** | **2.32** | **no** |
| **SAMA 70524.R2** | **Right** | **2** | **2.02** | **1.90** | **0.98** | **2.237** | **2.28** | **no** |
| SAMA 70524.R3 | Right | 3 | 2.23 | 1.74 | 0.99 | 2.172 | 2.19 | yes |
| SAMA 70524.R4 | Right | 4 | 1.85 | 1.37 | 0.81 | 1.985 | 2.47 | yes |
| SAMA 70524.R5 | Right | 5 | 1.54 | 1.36 | 0.73 | 1.849 | 2.55 | yes |
