# Supplementary material for: Neutron scanning reveals unexpected complexity in the enamel thickness of an herbivorous Jurassic reptile
Source: J R Soc Interface. 2018 Jun 13;15(143):20180039. doi: 10.1098/rsif.2018.0039 (PMC6030635; doi:10.1098/rsif.2018.0039)
Supplement: SI Table 2 [file rsif20180039supp4.docx]

**SI Table 2.** Plots constructed based on the experiemental results of Barani et al. (2012 [74]: fig. 3).

| **h/R** | **intercept** | **radial** | **intercept2** | **margin** | **radial coeff** | **margin coeff** |
| --- | --- | --- | --- | --- | --- | --- |
| 0.5 | 0.19 | 1.000 | 0.15 | 1.000 | 6.000 | 7.000 |
| 1.0 | 0.30 | 1.579 | 0.29 | 1.933 | 9.474 | 13.533 |
| 2.0 | 0.53 | 2.789 | 0.65 | 4.333 | 16.737 | 30.333 |
| 3.0 | 0.78 | 4.105 | 0.90 | 8.378 | 24.632 | 58.644 |
