# Supplementary material for: Neutron scanning reveals unexpected complexity in the enamel thickness of an herbivorous Jurassic reptile
Source: J R Soc Interface. 2018 Jun 13;15(143):20180039. doi: 10.1098/rsif.2018.0039 (PMC6030635; doi:10.1098/rsif.2018.0039)
Supplement: SI Table 3 [file rsif20180039supp5.docx]

### SI Table 3. Predicted critical load for the unworn teeth of *Sphenodon*. Three unworn dentary teeth are used from specimen SAMA 70524. Toughness of tuatara enamel is reported to range between 210000 and 32000 Yilmaz (et al. 2014, [38]). The lower bound relates to radial cracks, an enamel toughness of 210000, and an enamel thickness of 0.11 mm whereas the upper bound relates to marginal cracks, an enamel toughness of 320000, and an enamel thickness of 0.13 mm (Carlson 1990; Kieser et al. 2011; [36, 37]). Colours correspond to those used in Figure 3 and SI Table 2.

| **Tooth** | **crack type** | **enamel thickness** | **mesiodistal tooth dimension (mm)** | **labiolingual dimension (mm)** | **diameter of the tooth (m)** | **h/R** | **constant** | **crown radius (m)** | **Enamel toughness (N m^1.5^)** | **predicted critical load (N)** |
| --- | --- | --- | --- | --- | --- | --- | --- | --- | --- | --- |
| SAMA 70524.L2 | radial | 0.11 | 1.87 | 1.64 | 0.001755 | 1.95 | 17 | 0.00088 | 210000 | **33** |
| SAMA 70524.R1 | radial | 0.11 | 1.74 | 1.82 | 0.00178 | 2.32 | 20 | 0.00089 | 210000 | 39 |
| SAMA 70524.R2 | radial | 0.11 | 2.02 | 1.90 | 0.00196 | 2.28 | 19.5 | 0.00098 | 210000 | 42 |
| SAMA 70524.L2 | radial | 0.13 | 1.87 | 1.64 | 0.001755 | 1.95 | 17 | 0.00088 | 210000 | 36 |
| SAMA 70524.R1 | radial | 0.13 | 1.74 | 1.82 | 0.00178 | 2.32 | 20 | 0.00089 | 210000 | 43 |
| SAMA 70524.R2 | radial | 0.13 | 2.02 | 1.90 | 0.00196 | 2.28 | 19.5 | 0.00098 | 210000 | 46 |
| SAMA 70524.L2 | radial | 0.11 | 1.87 | 1.64 | 0.001755 | 1.95 | 17 | 0.00088 | 320000 | 50 |
| SAMA 70524.R1 | radial | 0.11 | 1.74 | 1.82 | 0.00178 | 2.32 | 20 | 0.00089 | 320000 | 60 |
| SAMA 70524.R2 | radial | 0.11 | 2.02 | 1.90 | 0.00196 | 2.28 | 19.5 | 0.00098 | 320000 | 64 |
| SAMA 70524.L2 | radial | 0.13 | 1.87 | 1.64 | 0.001755 | 1.95 | 17 | 0.00088 | 320000 | 54 |
| SAMA 70524.R1 | radial | 0.13 | 1.74 | 1.82 | 0.00178 | 2.32 | 20 | 0.00089 | 320000 | 65 |
| SAMA 70524.R2 | radial | 0.13 | 2.02 | 1.90 | 0.00196 | 2.28 | 19.5 | 0.00098 | 320000 | 70 |
| SAMA 70524.L2 | margin | 0.11 | 1.87 | 1.64 | 0.001755 | 1.95 | 34 | 0.00088 | 210000 | 66 |
| SAMA 70524.R1 | margin | 0.11 | 1.74 | 1.82 | 0.00178 | 2.32 | 42 | 0.00089 | 210000 | 82 |
| SAMA 70524.R2 | margin | 0.11 | 2.02 | 1.90 | 0.00196 | 2.28 | 40 | 0.00098 | 210000 | 86 |
| SAMA 70524.L2 | margin | 0.13 | 1.87 | 1.64 | 0.001755 | 1.95 | 34 | 0.00088 | 210000 | 71 |
| SAMA 70524.R1 | margin | 0.13 | 1.74 | 1.82 | 0.00178 | 2.32 | 42 | 0.00089 | 210000 | 90 |
| SAMA 70524.R2 | margin | 0.13 | 2.02 | 1.90 | 0.00196 | 2.28 | 40 | 0.00098 | 210000 | 94 |
| SAMA 70524.L2 | margin | 0.11 | 1.87 | 1.64 | 0.001755 | 1.95 | 34 | 0.00088 | 320000 | 100 |
| SAMA 70524.R1 | margin | 0.11 | 1.74 | 1.82 | 0.00178 | 2.32 | 42 | 0.00089 | 320000 | 125 |
| SAMA 70524.R2 | margin | 0.11 | 2.02 | 1.90 | 0.00196 | 2.28 | 40 | 0.00098 | 320000 | 132 |
| SAMA 70524.L2 | margin | 0.13 | 1.87 | 1.64 | 0.001755 | 1.95 | 34 | 0.00088 | 320000 | 109 |
| SAMA 70524.R1 | margin | 0.13 | 1.74 | 1.82 | 0.00178 | 2.32 | 42 | 0.00089 | 320000 | 136 |
| SAMA 70524.R2 | margin | 0.13 | 2.02 | 1.90 | 0.00196 | 2.28 | 40 | 0.00098 | 320000 | **143** |
