# Supplementary material for: Neutron scanning reveals unexpected complexity in the enamel thickness of an herbivorous Jurassic reptile
Source: J R Soc Interface. 2018 Jun 13;15(143):20180039. doi: 10.1098/rsif.2018.0039 (PMC6030635; doi:10.1098/rsif.2018.0039)
Supplement: SI Table 4 [file rsif20180039supp6.docx]

### SI Table 4. The 16-bit grayscale values (as given by Avizo) that typically represent different tooth components within the two datasets. The greyscale values from the X-ray dataset are proportional to raw attenuation by x (0.15/2^16^)+0.015 and those from the neutron dataset are proportional to raw attenuation by x 0.000028885.

| Material | X-ray dataset | Neutrons |
| --- | --- | --- |
| Enamel | 1800 to ~28000 | 18000 to 36000 |
| Dentine | ~25000 or more | ~35500 to 55000 |
| Pulp | 15000 to 30000 | 8000 to 32000 |
