# Supplementary material for: Neutron scanning reveals unexpected complexity in the enamel thickness of an herbivorous Jurassic reptile
Source: J R Soc Interface. 2018 Jun 13;15(143):20180039. doi: 10.1098/rsif.2018.0039 (PMC6030635; doi:10.1098/rsif.2018.0039)
Supplement: SI Table 5 [file rsif20180039supp7.docx]

### SI Table 5. Predicted critical load for the unworn teeth of *Eilenodon*. The lower bound relates to radial cracks and uses a h/R of 1.9 whereas the upper bound relates to marginal cracks and uses a h/R of 2.1. Toughness of tuatara enamel is reported to range between 210000 and 32000 (Yilmaz et al. 2014 [38]).

| **Tooth** | **crack type** | **enamel thickness** | **mesiodistal tooth dimension (mm)** | **labiolingual dimension (mm)** | **diameter of the tooth (m)** | **h/R** | **constant** | **crown radius (m)** | **Enamel toughness (N m^1.5^)** | **predicted critical load (N)** |
| --- | --- | --- | --- | --- | --- | --- | --- | --- | --- | --- |
| DMNH EPV.10685.1 | radial | 0.20 | 3.28 | 4.70 | 0.003399 | 1.9 | 17 | 0.0002 | 210000 | **101** |
| DMNH EPV.10685.1 | radial | 0.20 | 3.28 | 4.70 | 0.003399 | 2.1 | 18 | 0.0002 | 210000 | 107 |
| DMNH EPV.10685.1 | radial | 0.20 | 3.28 | 4.70 | 0.003399 | 1.9 | 17 | 0.0002 | 320000 | 153 |
| DMNH EPV.10685.1 | radial | 0.20 | 3.28 | 4.70 | 0.003399 | 2.1 | 18 | 0.0002 | 320000 | 163 |
| DMNH EPV.10685.1 | margin | 0.20 | 3.28 | 4.70 | 0.003399 | 1.9 | 34 | 0.0002 | 210000 | 201 |
| DMNH EPV.10685.1 | margin | 0.20 | 3.28 | 4.70 | 0.003399 | 2.1 | 36 | 0.0002 | 210000 | 213 |
| DMNH EPV.10685.1 | margin | 0.20 | 3.28 | 4.70 | 0.003399 | 1.9 | 34 | 0.0002 | 320000 | 307 |
| DMNH EPV.10685.1 | margin | 0.20 | 3.28 | 4.70 | 0.003399 | 2.1 | 36 | 0.0002 | 320000 | **325** |
